# Dynamic detection and reversal of myocardial ischemia using an artificially intelligent bioelectronic medicine

**DOI:** 10.1126/sciadv.abj5473

**Published:** 2022-01-05

**Authors:** Patrick D. Ganzer, Masoud S. Loeian, Steve R. Roof, Bunyen Teng, Luan Lin, David A. Friedenberg, Ian W. Baumgart, Eric C. Meyers, Keum S. Chun, Adam Rich, Allison L. Tsao, William W. Muir, Doug J. Weber, Robert L. Hamlin

**Affiliations:** 1 Medical Devices and Neuromodulation, Battelle Memorial Institute, 505 King Ave., Columbus, OH 43201, USA.; 2 Department of Biomedical Engineering, University of Miami, 1320 S Dixie Hwy., Coral Gables, FL 33146, USA.; 3 The Miami Project to Cure Paralysis, University of Miami, 1095 NW 14th Terrace #48, Miami, FL 33136, USA.; 4 QTest Labs, 6456 Fiesta Dr., Columbus, OH 43235, USA.; 5 Health Analytics, Battelle Memorial Institute, 505 King Ave., Columbus, OH 43201, USA.; 6 Cardiovascular Section, Department of Medicine, VA Boston Healthcare System, Boston, MA 02130, USA.; 7 Cardiovascular Division, Department of Medicine, Brigham and Women’s Hospital, Boston, MA 02115, USA.; 8 College of Veterinary Medicine, Lincoln Memorial University, 6965 Cumberland Gap Parkway, Harrogate, TN 37752, USA.; 9 Department of Mechanical Engineering and Neuroscience, Carnegie Mellon University, 5000 Forbes Ave., Pittsburgh, PA 15213, USA.; 10 Department of Veterinary Biosciences, The Ohio State University, 1900 Coffey Road, Columbus, OH 43201, USA.

## Abstract

Myocardial ischemia is spontaneous, frequently asymptomatic, and contributes to fatal cardiovascular consequences. Importantly, myocardial sensory networks cannot reliably detect and correct myocardial ischemia on their own. Here, we demonstrate an artificially intelligent and responsive bioelectronic medicine, where an artificial neural network (ANN) supplements myocardial sensory networks, enabling reliable detection and correction of myocardial ischemia. ANNs were first trained to decode spontaneous cardiovascular stress and myocardial ischemia with an overall accuracy of ~92%. ANN-controlled vagus nerve stimulation (VNS) significantly mitigated major physiological features of myocardial ischemia, including ST depression and arrhythmias. In contrast, open-loop VNS or ANN-controlled VNS following a caudal vagotomy essentially failed to reverse cardiovascular pathophysiology. Last, variants of ANNs were used to meet clinically relevant needs, including interpretable visualizations and unsupervised detection of emerging cardiovascular stress. Overall, these preclinical results suggest that ANNs can potentially supplement deficient myocardial sensory networks via an artificially intelligent bioelectronic medicine system.

## Teaser

Potentially damaging “heart stress” is reversed using reactive nerve stimulation controlled by artificial intelligence.

## INTRODUCTION

Cardiovascular disease is responsible for a staggering ~25 to 30% of mortality worldwide (*1*). One prominent attribute of cardiovascular disease is myocardial ischemia, caused by a decrease in myocardial oxygen supply and/or an increase in myocardial oxygen demand (*2*–*4*). Treating myocardial ischemia can reduce rates of myocardial injury, myocardial infarction, and death (*5*–*8*). Unfortunately, treating myocardial ischemia is accompanied by several major challenges.

Roughly 75% of ischemic episodes are asymptomatic (i.e., subclinical ischemia), where the heart can be irreversibly damaged without conscious awareness (*8*–*11*). This extensively complicates the detection and thus appropriate treatment of myocardial ischemia and clearly shows that myocardial sensory networks (*12*, *13*) are considerably deficient at detecting myocardial ischemia. Furthermore, myocardial ischemia can occur at random throughout the day (*8*, *9*, *14*), complicating its detection and treatment. Supplementing deficient myocardial sensory networks represents a promising approach to more effectively detect and potentially reverse myocardial ischemia.

In this preclinical study, we assessed the hypothesis that artificial neural networks (ANNs) can supplement deficient myocardial sensory networks (*8*, *12*, *13*) to detect, and even help correct, myocardial ischemia. To this end, we used an ANN that rapidly decodes events of spontaneous myocardial ischemia and responsively triggers therapeutic closed-loop vagus nerve stimulation (VNS). Responsive closed-loop VNS may be an effective bioelectronic medicine for reversing ischemia-mediated elevations in chronotropy, afterload, and myocardial oxygen demand (*15*–*17*).

Although promising, implementing an ANN-controlled bioelectronic medicine for myocardial ischemia is difficult for several reasons. Events of myocardial ischemia are physiologically variable, within and across subjects (*8*, *18*, *19*). Furthermore, nonischemic states have electrophysiological characteristics similar to myocardial ischemia (e.g., cardiac valve dysfunction, repolarization abnormalities, or an electrolyte imbalance) (*8*, *20*–*22*). Therefore, detecting myocardial ischemia is complicated by significant physiological feature variability and off-target states.

Most bioelectronic medicines use preprogrammed open-loop stimulation schedules. However, a closed-loop bioelectronic medicine that selectively responds when needed can optimize therapeutic efficacy (*23*–*26*). In addition, myocardial ischemia occurs throughout the day at random (*8*, *9*, *14*). Therefore, an effective bioelectronic treatment for myocardial ischemia may need to leverage responsive closed-loop control, where on-demand stimulation is autonomously triggered when needed for benefit.

Last, artificial intelligence (AI) is becoming a powerful tool in medicine. Importantly, AI-enabled medicines must be easily interpretable for widespread adoption (*27*, *28*). AI interpretability can be enhanced using visualizations. However, it can be challenging to create interpretable visualizations of both high-dimensional data and complex algorithm decisions (*27*, *29*). Furthermore, disease pathophysiology and recorded data are always changing; over time, subjects can experience new forms of cardiovascular stress, and new pathophysiological states may emerge (*14*, *30*). Therefore, future AI-enabled medicines will need to be both interpretable and adaptive to physiological changes.

## RESULTS

### Inducing acute myocardial ischemia

**Fig. 1.**
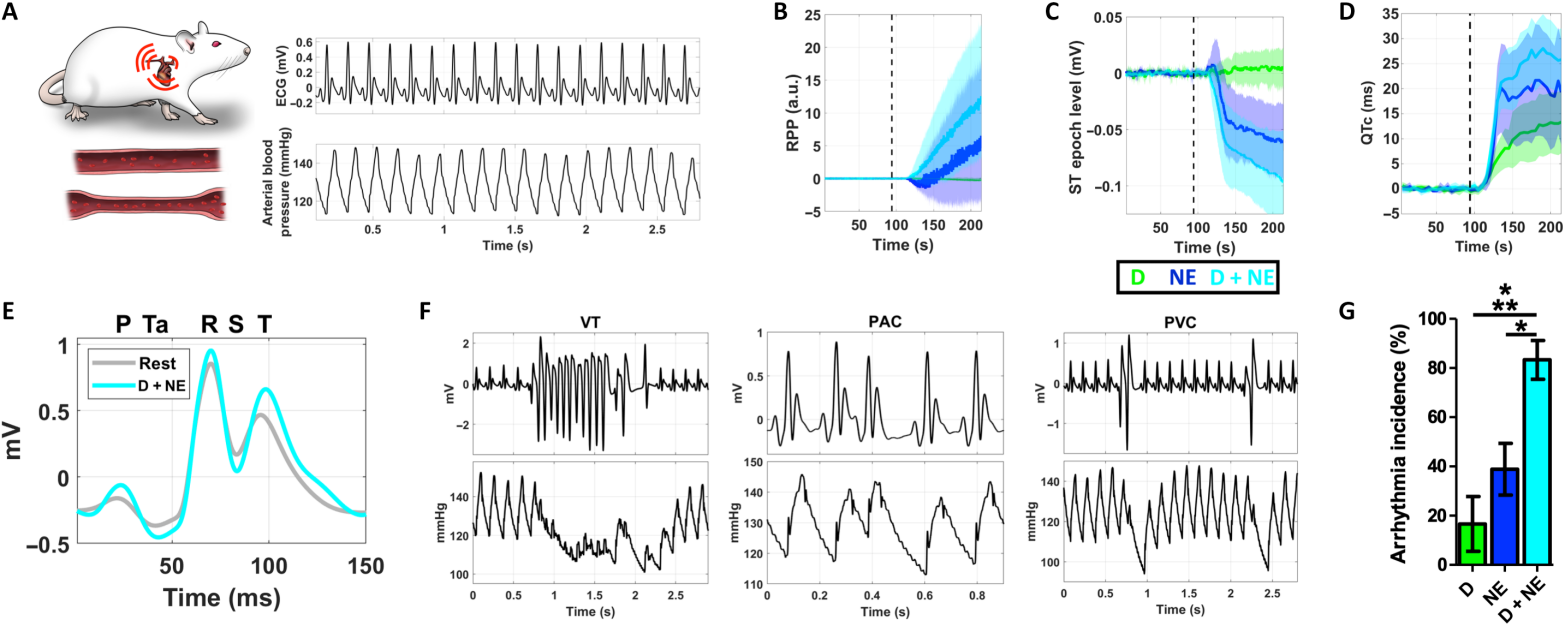
Inducing acute myocardial ischemia in vivo. (**A**) Cartoon of rat experiments for inducing cardiovascular stress and myocardial ischemia (heart/tachycardia cartoon, top left; blood vessel/vasoconstriction cartoon, bottom left; electrocardiogram (ECG) waveforms, top right; arterial blood pressure waveforms, bottom right). Rate-pressure product (RPP) (**B**), ST epoch level (**C**), and QTc (**D**) were differentially modulated following a dobutamine (D, green), norepinephrine (NE, blue), or combined dobutamine and norepinephrine (D + NE, cyan) infusion, indicative of cardiovascular stress and acute myocardial ischemia (time series include lighter shaded regions, ±95% confidence intervals; vertical black dashed line, start of the respective infusion). (**E**) Representative cycle averaged ECGs during rest (gray) or D + NE–induced myocardial ischemia (cyan). Note the pronounced suppression of ECG epochs during both diastole and systole, indicative of ischemic currents (relative P, Ta, R, S, and T ECG wave time points shown at the top of the panel). (**F**) Several arrhythmia types occurred during acute cardiovascular stress and myocardial ischemia, including VT, PAC, and PVC (top, ECG waveforms; bottom, arterial blood pressure waveforms). (**G**) Arrhythmia incidence progressively increased across infusion type, reaching a maximum of ~83% for the D + NE combination (* indicates different at *P* < 0.05; ** indicates different at *P* < 0.001). Data presented are means ± SEM. a.u., arbitrary units.

Myocardial ischemia is associated with enhanced catecholamine tone, increased afterload, and changes to the myocardial oxygen supply/demand ratio commonly lasting 30 s or more (*4*, *8*, *10*, *11*, *31*, *32*). We modeled these attributes of myocardial ischemia using relatively high dose infusions of dobutamine and/or norepinephrine in rats (2 to 3 μg kg^−1^ min^−1^ each), targeting precipitous activation of adrenergic α and β receptors. Our overall model was motivated by multiple factors: (i) High-dose infusions of catecholamines are well known to precipitously disrupt myocardial energetic imbalance, acutely leading to electrocardiographic signatures of ischemia and, eventually, distributed myocardial cell death (*33*–*36*); (ii) high-dose activation of adrenergic α and β receptors has been shown to reduce coronary blood flow and induce coronary vasospasm, therefore facilitating myocardial ischemia (*35*–*38*); (iii) acute infusions allow for cardiovascular event control and repeated events within an animal. We used three types of infusions (schematic of experimental interfaces in fig. S1A): (i) dobutamine alone (D), (ii) norepinephrine alone (NE), or (iii) dobutamine and norepinephrine combined (D + NE). Infusion protocols consisted of an initial rest period followed by a 2-min infusion period (infusion start, vertical dashed line, Fig. 1, B to E).

Each infusion type differentially affected traditional physiological features of cardiovascular stress and myocardial ischemia, including rate-pressure product (RPP, an index of myocardial oxygen consumption; Fig. 1B) (*39*), ST epoch level (a classic electrophysiological feature of subendocardial ischemia; Fig. 1C) (*40*), and corrected QT (QTc) prolongation (using Bazett’s formula; an electrophysiological abnormality occurring during acute myocardial ischemia, related to proarrhythmic states; Fig. 1D) (*41*, *42*). The combined D + NE infusion tended to have the largest effect. A representative averaged electrocardiogram (ECG) is shown before (rest, gray; Fig. 1E) and during D + NE–induced myocardial ischemia (D + NE, cyan; Fig. 1E). Electrophysiological correlates of decreased myocardial membrane potential and ischemic currents (*40*, *43*, *44*) were observed during both systole (during QRS) and diastole (during the ST and Ta epochs). An increased RPP was significantly correlated to both ST epoch level depression (*R* = −0.63; *P* < 0.001) and QTc prolongation across all infusions (*R* = 0.52; *P* < 0.001). Therefore, higher myocardial oxygen consumption was associated with both increased subendocardial ischemia and prolonged ventricular action potential duration. Last, several arrhythmia types occurred [including ventricular tachycardias (VTs), premature atrial contractions (PACs), and premature ventricular contractions (PVCs); Fig. 1F], with D + NE evoking the highest arrhythmia incidence (*F*[2,24] = 10.4, *P* < 0.001; Fig. 1G). Overall, these results demonstrate the ability to induce cardiovascular stress and acute myocardial ischemia, with observed physiological feature changes similar to human myocardial ischemia (*4*, *8*, *10*, *11*, *31*, *32*, *41*, *42*).

### Creating features for cardiovascular state decoding

**Fig. 2.**
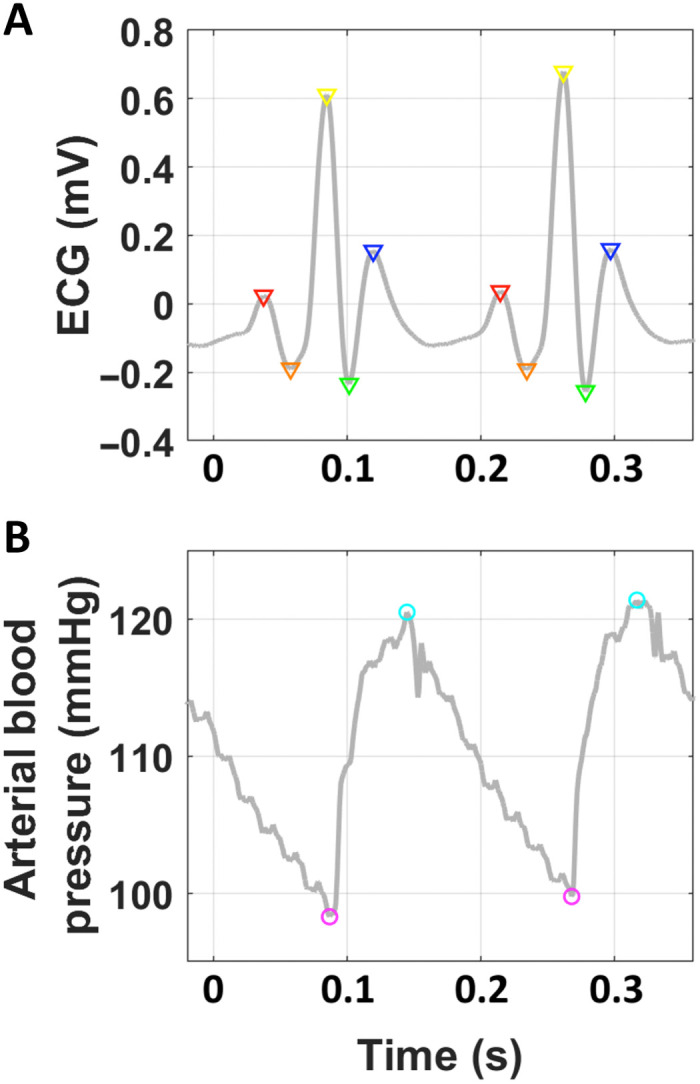
Schematic of cardiovascular feature components. All 13 cardiovascular features (shown in Fig. 3) were derived from the ECG (**A**) or arterial blood pressure (**B**) signals. Across wave cycles, we identified correlates of the P wave (red triangles, atrial depolarization), Ta wave (orange triangles, atrial repolarization), R wave (yellow triangles, ventricular depolarization), S wave (green triangles, nadir between ventricular depolarization and repolarization), T wave (blue triangles, ventricular repolarization), diastolic blood pressure (magenta circles), and systolic blood pressure (cyan circles). Breath rate was derived from the linear envelope of the blood pressure signal. The 13-element feature vector was calculated every 100 ms and averaged over a 4-s sliding window. Please see the “Online cardiovascular signal conditioning and feature extraction” section in Materials and Methods for more details on feature extraction.

We next created a broader set of features from the ECG and blood pressure signals for state decoding and target ischemia detection (ECG feature schematic in Fig. 2A; blood pressure feature schematic in Fig. 2B). The broader 13-element feature vector further quantified several physiological parameters, including ECG segment durations (milliseconds), relative ECG wave point levels (millivolts), blood pressures during diastole and systole [millimeters of mercury (mmHg)], and breath rate. Changes in features were assessed for D, NE, and D + NE, quantified with respect to baseline levels (i.e., ∆ relative to baseline).

**Fig. 3.**
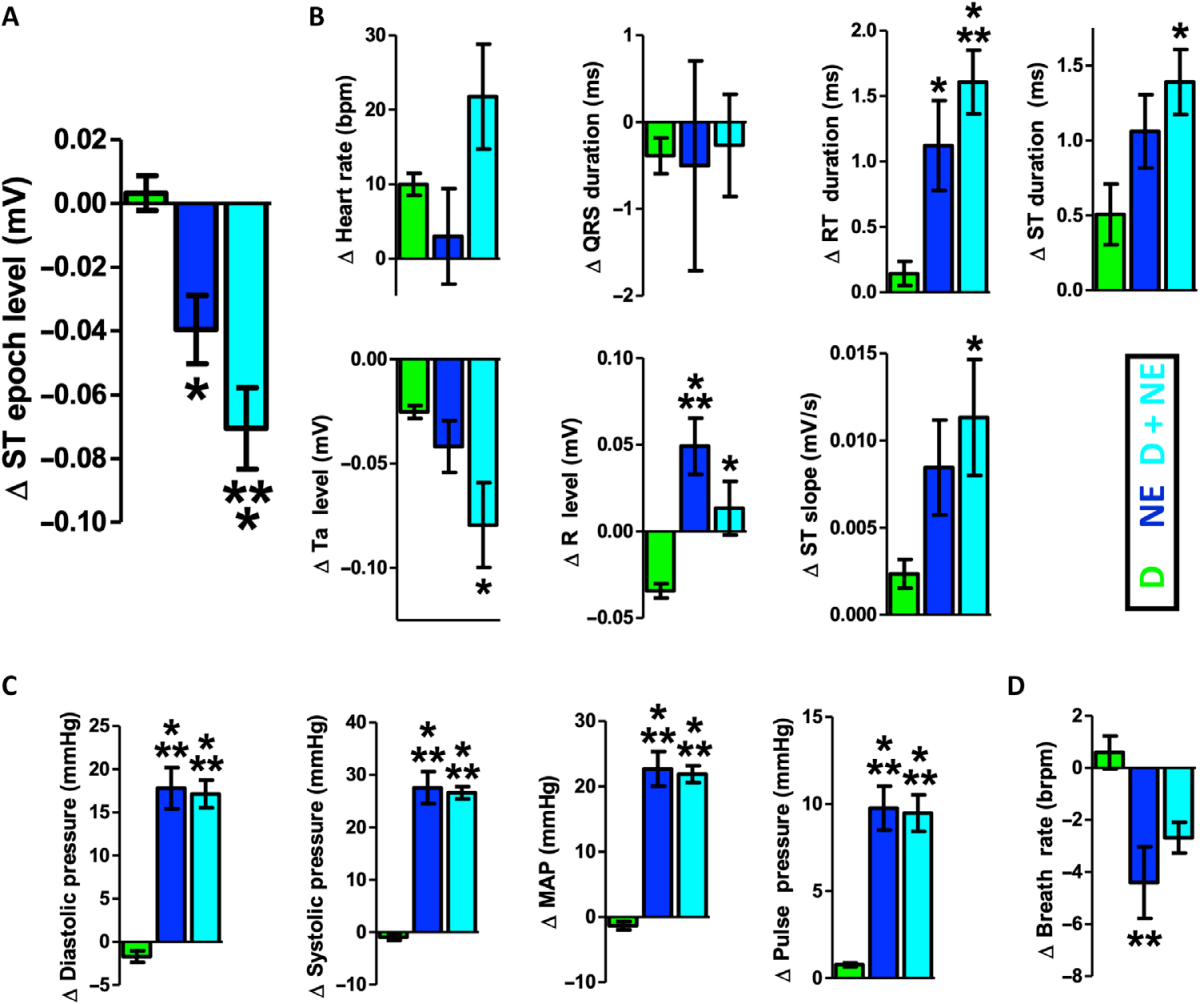
Cardiovascular feature changes during induction of cardiovascular stress and myocardial ischemia. A more detailed 13-element cardiovascular feature vector was created for eventual decoding of cardiovascular state. Dobutamine (D, green), norepinephrine (NE, blue), or a combination of dobutamine and norepinephrine (D + NE, cyan) induced significant changes to the clinically relevant ST level (**A**) and also modified other features related to the ECG (**B**), blood pressure (**C**), and pulmonary function (**D**). Notably, D + NE maximally affected several cardiovascular features (* indicates different from D at *P* < 0.05; ** indicates different from D at *P* < 0.01; *** indicates different from D at *P* < 0.001). This 13-element feature vector was next used for decoding cardiovascular stress and myocardial ischemia. Data presented are means ± SEM.

Although several cardiovascular features are related to myocardial ischemia, ST changes are a clinically accepted “gold standard” physiological feature of myocardial ischemia. Importantly, ST epoch level significantly depressed, indicative of subendocardial ischemia (Fig. 3A; complimenting the ST epoch–level time series shown in Fig. 1C) (*40*, *43*, *44*). Additional physiological attributes of cardiovascular stress and myocardial ischemia were also observed across the added features (*10*, *32*, *40*, *43*, *44*), including increases in afterload, decreases in myocardial conduction velocity, and depression of other ECG wave points indicative of ischemic currents [Fig. 3B, additional ECG features; Fig. 3C, blood pressure features; Fig. 3D, pulmonary feature; Table 1, omnibus analysis of variance (ANOVA) results]. The combined infusion of D + NE also had a maximal effect on this broader set of features compared to D or NE. These results demonstrate the more distributed impact of catecholamines on broader features of cardiovascular stress and myocardial ischemia.

**Table 1.** Omnibus ANOVA results.

	**Omnibus ANOVA results**
**∆ Feature 1 (ST epoch level)**	*F*[2,24] = 13.3, *P* < 0.001
**∆ Feature 2 (heart rate)**	*F*[2,24] = 2.8, *P* = 0.07
**∆ Feature 3 (QRS duration)**	*F*[2,24] = 0.02, *P* = 0.97
**∆ Feature 4 (RT duration)**	*F*[2,24] = 8.9, *P* < 0.01
**∆ Feature 5 (ST duration)**	*F*[2,24] = 4, *P* < 0.05
**∆ Feature 6 (Ta level)**	*F*[2,24] = 4, *P* < 0.05
**∆ Feature 7 (R level)**	*F*[2,24] = 10.1, *P* < 0.001
**∆ Feature 8 (ST slope)**	*F*[2,24] = 3.2, *P* < 0.05
**∆ Feature 9 (diastolic pressure)**	*F*[2,24] = 44.2, *P* < 0.001
**∆ Feature 10 (systolic pressure)**	*F*[2,24] = 71.3, *P* < 0.001
**∆ Feature 11 (mean arterial** **pressure)**	*F*[2,24] = 60.7, *P* < 0.001
**∆ Feature 12 (pulse pressure)**	*F*[2,24] = 28.9, *P* < 0.001
**∆ Feature 13 (breath rate)**	*F*[2,24] = 7.3, *P* < 0.01

Physiological features of myocardial ischemia can be variable within and across individuals (e.g., consistency of episodes and overall electrocardiographic feature variability) (*18*, *19*). Furthermore, seemingly separate cardiovascular states can exhibit highly correlated physiological features and thus statistically overlap (*8*, *20*–*22*, *45*). Therefore, both physiological variability and correlation across states should be attributes of a myocardial ischemia model. The variability of myocardial ischemia represents a clinically relevant challenge for event detection and decoding.

The cardiovascular feature data (from Fig. 3) exhibited variability and disorder comparable to human cardiovascular data recorded in either the intensive care unit (fig. S2A) (*46*, *47*) or during ambulatory episodes of myocardial ischemia (fig. S2B) (*46*, *48*, *49*). Furthermore, there was a significant correlation between NE and D + NE, even though they are distinct and separate cardiovascular stress states (fig. S2C). These findings demonstrate that the recorded cardiovascular features importantly model the variability and state overlap seen during human cardiovascular stress and myocardial ischemia, a clinically relevant challenge for cardiovascular state decoding.

### Decoding complex cardiovascular states using an ANN

Myocardial sensory networks are largely incapable of detecting myocardial ischemia (~75% of episodes are asymptomatic and therefore subclinical) (*8*–*10*). An ANN may be able to supplement deficient myocardial sensory networks to reliably detect, and even help correct, myocardial ischemia. We developed an ANN architecture to decode cardiovascular states during cardiovascular stress and myocardial ischemia, composed of both a hidden dense layer and a hidden long short-term memory (LSTM) layer (four total layers; schematic in fig. S3A; see the “Decoding myocardial ischemia using an ANN” section in Materials and Methods for more details). An LSTM layer was incorporated to detect long-term dependencies across time in the cardiovascular data and potentially enhance decoding performance (*50*, *51*). The output of the ANN is a continuous prediction score across the four states: rest (no drug infused), D, NE, or D + NE (example decoder outputs during a D + NE infusion in fig. S3B).

**Fig. 4.**
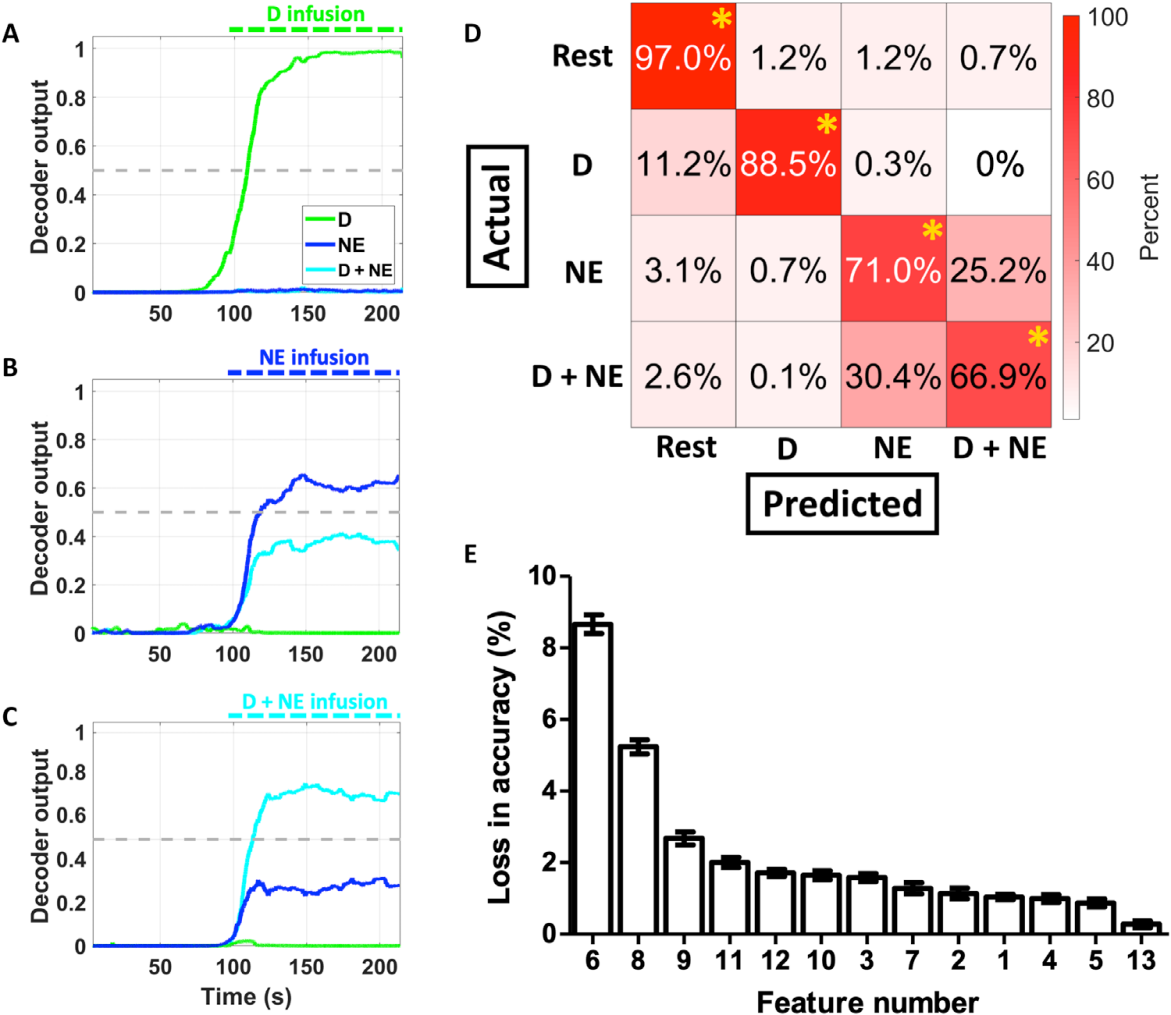
The ANN accurately classifies cardiovascular stress states and is significantly affected by the removal of features related to cardiac electrophysiology and vascular resistance. The ANN was challenged to decode complex cardiovascular state changes (cardiovascular feature variability and state overlap assessment in fig. S2) across a total of four classes: rest, D, NE, or D + NE. Continuous decoder outputs are shown for the infusion of D (**A**), NE (**B**), or D + NE (**C**). The ANN performed with a high overall accuracy (~92%) and sensitivity (~86%) [confusion matrix showing average performance values: (**D**) * indicates above chance at *P* < 0.001]. (**E**) The removal of features related to ECG ischemic currents (features 6 and 8) or blood pressure (features 9 to 12) led to the largest losses in decoding accuracy. These results show that an ANN can accurately decode cardiovascular states despite significant cardiovascular feature variability and state overlap. Data presented are means ± SEM.

Despite significant feature variability and state overlap, the ANN decoded cardiovascular state with high overall accuracy (~92%, *F*[3,36] = 163.5, *P* < 0.001; Fig. 4 and fig. S3C). Replacing the LSTM layer with a normal dense layer removed the network’s ability to assess long-term dependencies in the signal, significantly decreasing accuracy (i.e., an ANN-NO-LSTM architecture; fig. S3C). The ANN also outperformed other classifiers such as a support vector machine (SVM) or a linear discriminant analysis (LDA) (fig. S3C).

The ANN exhibited an overall sensitivity of ~86% (example decoder outputs in Fig. 4, A to C; confusion matrix showing average performance values in Fig. 4D). Although the ANN had an overall accuracy of ~92%, the most common misclassification was between the NE and D + NE classes (potentially due to their high degree of variability and correlation, as shown in Figs. 1 and 3 and fig. S2C). Features related to ECG wave point depression, ischemic currents, and vascular resistance were the most important for ANN decoding performance (Fig. 4E and Table 2). Last, a fixed ANN trained on subsets of the data robustly generalized to testing days spread out over several months and different animals (fig. S4). Overall, these results show that ANNs can robustly decode complex cardiovascular states to supplement deficient myocardial sensory networks.

**Table 2.** Feature number and name, ranked according to loss in accuracy (i.e., highest loss to lowest loss).

**Feature** **number**	**6**	**8**	**9**	**11**	**12**	**10**	**3**	**7**	**2**	**1**	**4**	**5**	**13**
**Feature** **name**	Ta level	ST slope	Diastolic pressure	Mean arterial pressure	Pulse pressure	Systolic pressure	QRS duration	R level	Heart rate	ST epoch level	RT duration	ST duration	Breath rate

### Responsive ANN-controlled VNS reverses myocardial ischemia

**Fig. 5.**
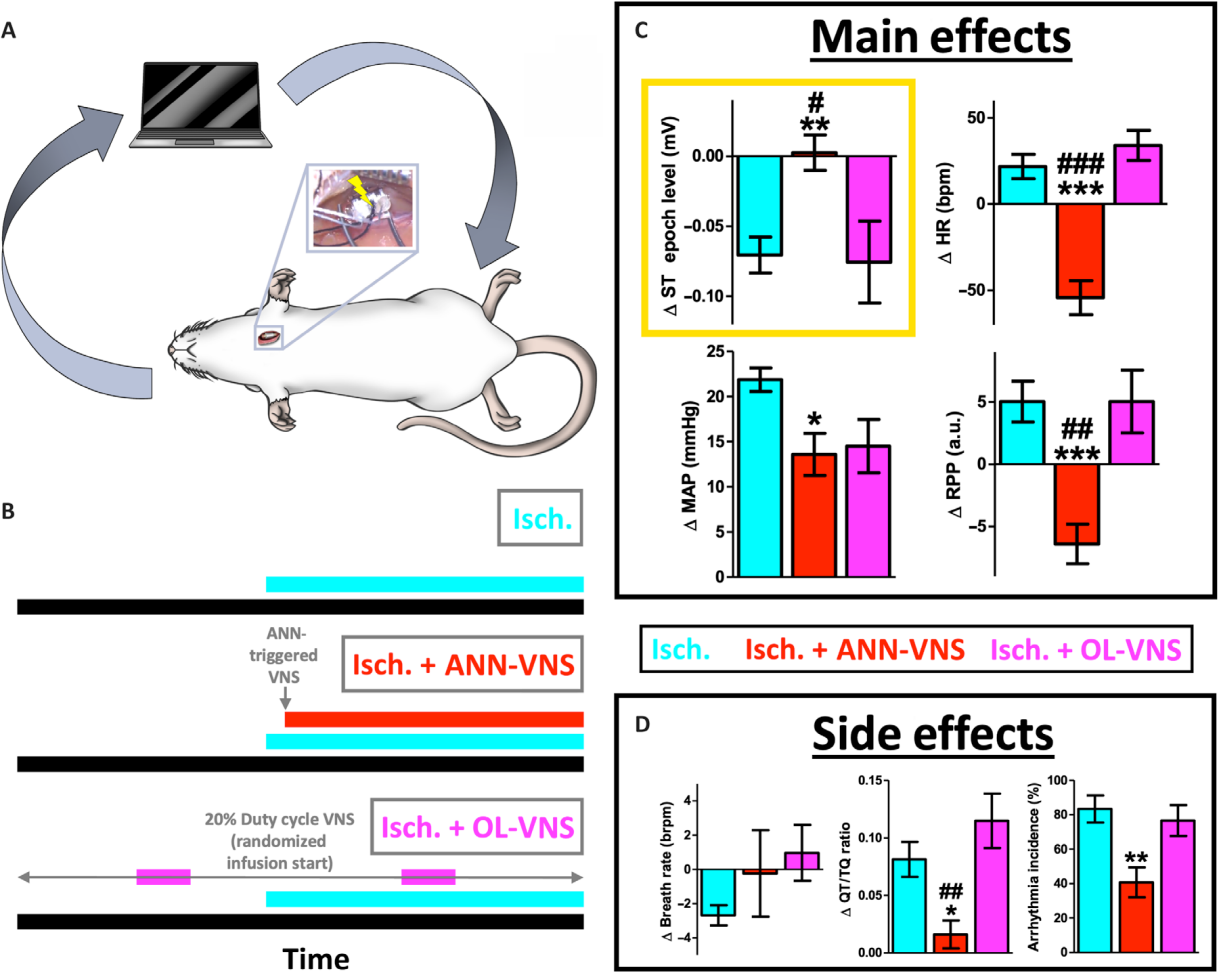
ANN-VNS reverses several pathophysiological features of myocardial ischemia. (**A**) The ANN was next used online in vivo for rapid detection of spontaneous myocardial ischemia and control of VNS (ANN-VNS; inset: left cervical vagus nerve and VNS cuff during implant). (**B**) We assessed the physiological features of cardiovascular stress and myocardial ischemia during either D + NE ischemia alone (cyan, Isch.), D + NE ischemia and closed-loop ANN-VNS (red, Isch. + ANN-VNS), or D + NE ischemia and open-loop VNS (magenta, Isch. + OL-VNS). (**C**) Only closed-loop ANN-VNS (red, Isch. + ANN-VNS) reversed several physiological features of myocardial ischemia, including ST epoch level (a gold standard electrocardiographic feature of subendocardial ischemia), heart rate, MAP (a correlate of afterload), and RPP (an index of myocardial oxygen consumption). Open-loop VNS (magenta, Isch. + OL-VNS) failed to reverse the physiological features of myocardial ischemia and was essentially no different from myocardial ischemia alone (cyan, Isch.) (different from Isch. at ***P < 0.001,**P < 0.01, or *P < 0.05; different from Isch. + OL-VNS at ###*P* < 0.001, ##*P* < 0.01, or #*P* < 0.05). (**D**) There were no significant differences in breath rate across groups. Only closed-loop ANN-VNS significantly mitigated side effects related to reentry risk (QT/TQ ratio) or arrhythmia incidence. These results demonstrate the ability of ANNs to supplement myocardial sensory networks and facilitate the reversal of spontaneous myocardial ischemia in vivo using a bioelectronic medicine. Data presented are means ± SEM.

Myocardial ischemia can cause irreversible heart damage if not treated rapidly. Therefore, beyond rapid detection alone, rapid correction is also needed. We next leveraged the ANN decoder to enable closed-loop VNS and potentially reverse myocardial ischemia (i.e., ANN-VNS; cartoon schematic in Fig. 5A). VNS can decrease chronotropy, afterload, and myocardial oxygen demand (*15*, *16*), all factors that are elevated during spontaneous myocardial ischemia (*3*, *4*, *32*). D + NE targets catecholamine receptors relevant for myocardial ischemia and has a maximal effect on the recorded features. Therefore, we targeted D + NE–induced myocardial ischemia for detection and potential correction, using ANN-VNS.

In real time and in vivo, the ANN detected spontaneous D + NE–induced myocardial ischemia with high overall accuracy (~94%; fig. S5A; average decoder outputs in fig. S5B), similar to offline performance (~92%). The ANN decoder output for D + NE–induced myocardial ischemia was also highly correlated with ST epoch level depression (*R* = 0.96, *P* < 0.001; fig. S5C). Therefore, the ANN performed with high accuracy in vivo and was synchronous with classic electrocardiographic evidence of myocardial ischemia.

ANN-VNS fully reversed ST epoch level depression, providing electrocardiographic evidence of myocardial ischemia mitigation (Fig. 5C, gold box, Isch. + ANN-VNS, red). ANN-VNS also reversed pathophysiological changes in heart rate, mean arterial pressure (MAP), and RPP (Fig. 5C, Isch. + ANN-VNS, red), compared to D + NE ischemia alone (Fig. 5C, Isch., cyan; heart rate: *F*[2,20] = 29.6, *P* < 0.001; MAP: *F*[2,20] = 5, *P* < 0.05; RPP: *F*[2,20] = 14.2, *P* < 0.001; ST epoch level: *F*[2,20] = 7.6, *P* < 0.01; full 13-element feature vector shown in fig. S6A; experimental schematic in Fig. 5B).

Open-loop VNS failed to reverse any major physiological features of D + NE–induced myocardial ischemia [Fig. 5C, Isch. + OL-VNS, magenta; open-loop VNS = 20% duty cycle, with a balanced amount of VNS compared to the closed-loop ANN-VNS paradigm, and parameters similar to previous human open-loop VNS studies; Table 1 (*52*, *53*); full 13-element feature vector shown in fig. S6B; experimental schematic in Fig. 5B, magenta]. There were no significant side effects across groups related to breath rate (breath rate: *F*[2,20] = 0.9, *P* = 0.41; Fig. 5D). Only ANN-VNS significantly mitigated side effects related to reentry risk (QT/TQ ratio: *F*[2,20] = 9.3, *P* < 0.01; Fig. 5D) (*54*) or arrhythmia incidence (arrhythmia incidence: *F*[2,20] = 6.8, *P* < 0.01; Fig. 5D) and therefore enhanced myocardial electrical stability. These findings demonstrate that preprogrammed open-loop VNS misses spontaneous myocardial ischemia that ANN-VNS is designed to respond to and correct. Overall, these preclinical results support the hypothesis that ANNs can supplement deficient myocardial sensory networks in a number of ways: not only via detection but also using bioelectronic control for correction of spontaneous cardiovascular pathophysiology.

Last, we performed a vagotomy caudal to the VNS site to, in part, examine the role of efferent vagal fiber activation. Caudal vagotomies blocked the major effects of ANN-VNS, indicating that the efferent vagal fibers are critical for the therapeutic effects of ANN-VNS (fig. S6C, orange). Last, all three VNS groups received an equivalent amount of VNS (*F*[2,14] = 1.3, *P* = 0.28; fig. S5D). These additional findings show that both the vagal fibers engaged and VNS timing (not necessarily VNS quantity) play critical roles in myocardial ischemia reversal.

### Detecting new emerging cardiovascular stress states using ANN autoencoders

Our next set of experiments addressed the need for decoding architectures to adapt as physiology changes. Over time, individuals can engage in new activities, and new forms of cardiovascular stress can emerge (*14*, *30*). A clinically deployed decoding architecture will eventually fail if it is not capable of detecting new emerging physiological states. We assessed techniques potentially capable of detecting new, unknown, and emerging cardiovascular stress states (emerging state/outlier detection review) (*55*). To model new unknown emerging stress, we used cardiovascular feature data recorded during a higher magnitude of cardiovascular stress and myocardial ischemia (i.e., at a higher dose level; emerging stress states: H-D, H-NE, and H-D + NE). A subset of the detection techniques used an ANN approach (i.e., autoencoders).

**Fig. 6.**
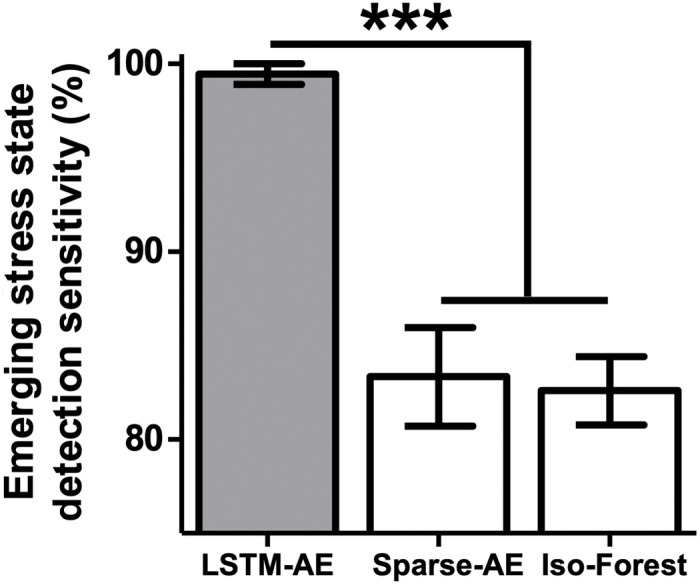
Detecting new emerging cardiovascular stress states. We implemented techniques for detecting new emerging stress states (new emerging stress states: high-dose versions of D, NE, and D + NE). LSTM-AEs significantly outperformed the Sparse-AE and Iso-Forest approaches (*** indicates different at *P* < 0.001). These results support the hypothesis that ANNs can also be used to detect new emerging stress states, as physiology evolves over time. Data presented are means ± SEM.

LSTM autoencoders (LSTM-AEs) detected new emerging stress states with a sensitivity of ~99%, even though the network was not exposed to these states during training (Fig. 6; *F*[2,27] = 26, *P* < 0.001; reconstruction loss distributions for known and unknown stress data in fig. S7; no significant differences for “known states,” i.e., D, NE, and D + NE, sensitivity across the three techniques, *F*[2,27] = 2.1, *P* = 0.13). Using sparse autoencoders (Sparse-AEs) removed the ability to assess long-term dependencies in the data, significantly decreasing emerging stress state detection performance (i.e., no LSTM components, a Sparse-AE; Fig. 6). The ANN-enabled LSTM-AE also outperformed the widely used isolation forest (Iso-Forest) technique (Fig. 6). These results further demonstrate how myocardial sensory networks can be supplemented with ANNs and suggest that ANNs can also potentially adapt to new emerging physiological changes.

### Enabling interpretable and adaptive AI: Visualizing emerging stress states within the “cardiovascular latent space” and unsupervised dissociation of different emerging stress types

AI-enabled medicines can suffer from a lack of interpretability, where data and/or algorithm decisions cannot be readily understood. AI-enabled medicines must be easily interpretable for widespread adoption (*27*, *28*). Visualizations are one solution for creating interpretable representations of both high-dimensional data and complex algorithm decisions.

**Fig. 7.**
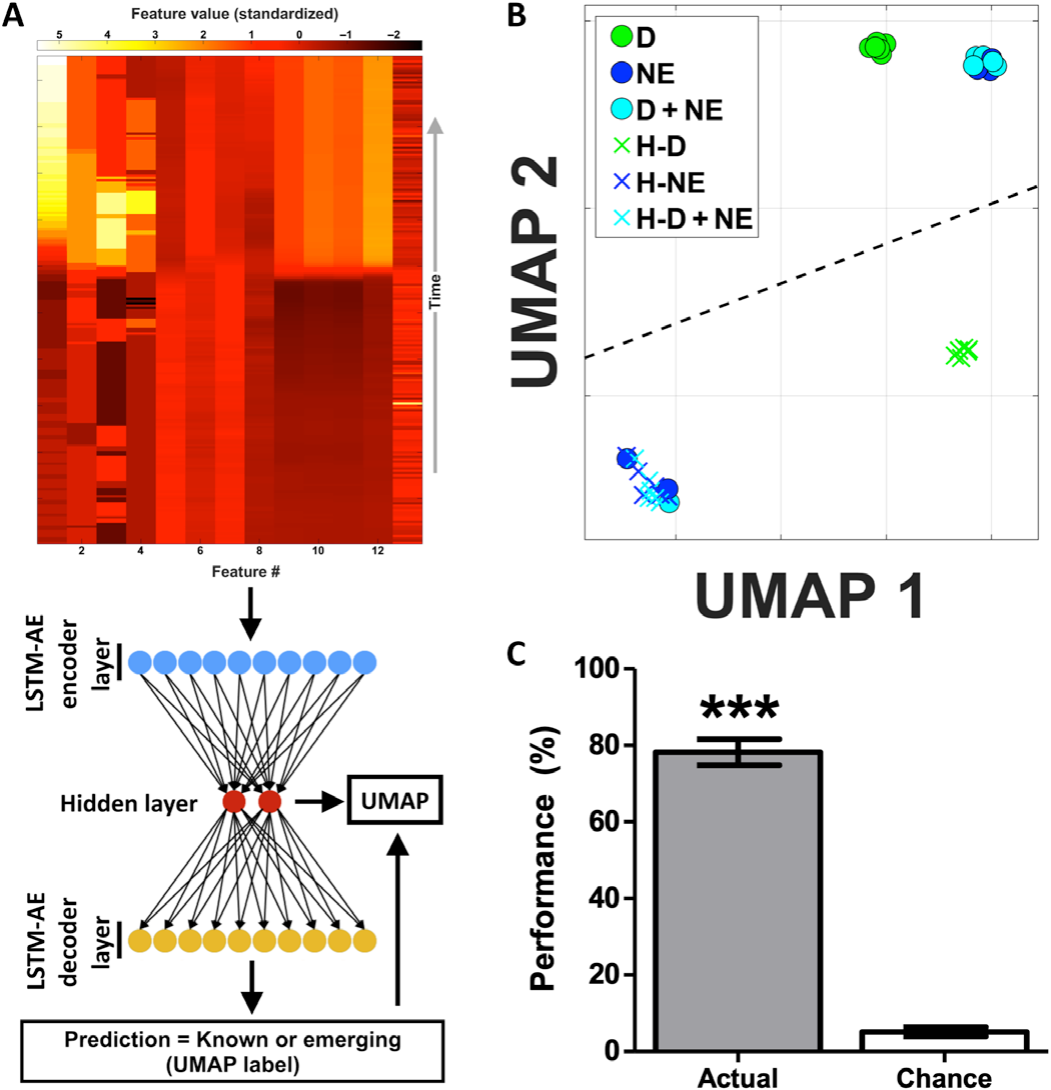
Leveraging the cardiovascular latent space for unsupervised identification of new emerging cardiovascular stress states. (**A**) Schematic of the emerging state detection architecture. For a given stress state observation, the feature matrix (top) is passed into the LSTM-AE (consisting of the encoder, hidden layer, and decoder components; LSTM-AE cartoon not to scale). Using the reconstruction loss, the LSTM-AE then predicts whether the given observation is a known or new emerging stress state. This prediction is then used as a label for UMAP dimensionality reduction and subsequent visualization of the LSTM-AE’s processes (see movie S1 for a representative video of these processes). (**B**) The hidden layer of the LSTM-AE is an uninterpretable 256-dimensional vector. We next generated an interpretable version of all known and unknown stress states using a combination of the LSTM-AE hidden layer and the dimensionality reduction technique UMAP (uninterpretable input, 256 dimensions; interpretable output, 2 dimensions). This cardiovascular latent space interestingly contained clustering of known (circles) and unknown emerging stress states (X’s), indicating that the cardiovascular latent space may also be useful for identifying different types of new emerging stress states (plotted data are representative of overall performance; black dashed line, linear decision boundary, calculated using an SVM). (**C**) We next combined the cardiovascular latent space with unsupervised clustering to potentially identify different types of new emerging stress states (via HDBSCAN). This fully unsupervised method achieved 78% performance when challenged to autonomously detect emerging stress states, performing well above chance performance levels (*** indicates different from chance at *P* < 0.001). Data presented are means ± SEM. Overall, these findings show that an ANN can help enable an interpretable and unsupervised emerging state detection architecture, relevant for a detection system that can adapt as physiology changes.

We next created an interpretable visualization of all known and new emerging stress states [using the LSTM-AE hidden layer and the dimensionality reduction technique uniform manifold approximation and projection (UMAP); Fig. 7A] (*56*). This architecture approach has recently achieved state-of-the-art performance converting complex high-dimensional data into interpretable representations (*57*). Across all stress states, the uninterpretable high-dimensional LSTM-AE hidden layer (256 dimensions) was transformed to an interpretable 2-dimensional representation (Fig. 7B, fig. S8, and movie S1). In this cardiovascular latent space, known and new emerging states formed clear clusters (Fig. 7B). Furthermore, known and new emerging stress states generally occupied separate regions of the cardiovascular latent space at ~85% accuracy, performing well above chance levels (*t*[18] = 7.1, *P*< 0.001; data from all 10 folds in fig. S8; assessing, the ability to separate the known and new emerging stress state clusters using a linear boundary). This interpretable visual information showcases the ability of ANNs to help meet clinical needs and create meaningful representations of complex high-dimensional cardiovascular data, even though the architecture has never been exposed to the new emerging cardiovascular stress state data.

Emerging state detection architectures should also be capable of autonomously identifying different types of emerging states, if multiple types exist. Unfortunately, this is exceedingly challenging, as the architecture cannot be exposed to one or multiple types of new emerging stress states during training. To address this challenge, our final analyses leveraged the LSTM-AE–enabled cardiovascular latent space combined with unsupervised clustering [unsupervised clustering method: hierarchical density-based spatial clustering of applications with noise (HDBSCAN)] (*58*). This fully unsupervised architecture achieved 78% performance when challenged to autonomously identify different types of emerging stress states, performing well above chance levels {Fig. 7C; i.e., unsupervised dissociation of varying combinations of H-D, H-NE, and H-D + NE; *t*[18] = 20.1, *P*< 0.001; performance metric = V-measure × 100% (*59*); V-measure is a well-studied metric to quantify clustering and detection capability; see fig. S9 for separate completeness, homogeneity, and performance values for all seven emerging stress state scenarios}. The architecture achieved this performance, in spite of unsupervised operation and notable out-of-sample generalization to multiple types of new emerging stress states. Overall, these results show that ANNs can further enable an interpretable unsupervised emerging state detection architecture, relevant for adapting to physiological changes over time.

## DISCUSSION

“It is hard to understand the biological strategy and hence development of a system providing the wild animal with hundreds of fibers exclusively designed for signaling unlikely coronary emergencies” [A. Malliani (*60*)].

In this proof-of-concept study, we demonstrate several ways ANNs can potentially supplement myocardial sensory networks (*12*, *13*). ANNs effectively decoded cardiovascular states with high accuracy, even though physiological features exhibited significant variability and state overlap, similar to human myocardial ischemia. Beyond detection alone, an ANN-enabled bioelectronic medicine reversed myocardial ischemia by reactively triggering VNS to reduce correlates of chronotropy, afterload, and myocardial oxygen demand. Preprogrammed open-loop VNS and ANN-VNS without efferent vagal fibers intact both failed to reverse myocardial ischemia, demonstrating the importance of VNS timing and vagal fibers engaged. Last, ANNs enabled clinically relevant interpretable visualizations and adaptive detection of emerging cardiovascular stress. This proof-of-concept study suggests that ANNs could supplement deficient myocardial sensory networks via an artificially intelligent bioelectronic medicine system to reduce myocardial ischemia.

### Supplementing deficient myocardial sensory networks with ANNs

It is exceedingly problematic that the leading cause of mortality worldwide, cardiovascular disease and myocardial ischemia, largely develops without conscious awareness. Approximately 75% of myocardial ischemia events are asymptomatic and therefore subclinical, known as “silent myocardial ischemia” (*8*–*10*). Furthermore, up to ~50% of myocardial infarctions (i.e., “heart attacks”) are also asymptomatic and happen without any sensation (*61*). These significant deficits in myocardial sensory networks (*12*, *13*) likely come from several sources.

First, deficient detection of myocardial ischemia may be due to evolutionary constraints. Several human-related factors that contribute to cardiovascular disease are relatively new from an evolutionary perspective, including consuming high-fat foods, smoking, and a sedentary lifestyle (*62*). Therefore, there may have been insufficient time to develop an effective cardiovascular pathophysiology detection system in humans, via evolutionary modifications to neural systems or other mechanisms (*60*, *62*, *63*).

Second, ischemia itself and other diseases contribute to deficient myocardial ischemia detection. Symptomatic ischemia, known as angina, only comprises ~25% of all ischemic events and is often misdiagnosed as off-target musculoskeletal pain, making it difficult to detect accurately (*8*, *64*). Even when angina occurs, subsequent ischemic events can be silenced and become subclinical, via desensitization of afferent signaling (known as “neural stunning”) (*8*). Several myocardial sensory network substrates may contribute to this, including specific afferent fiber systems, molecular messengers, and overall neurophysiological dynamics (*8*, *12*, *13*). Lastly, cardiovascular disease can accompany other disorders such as diabetes. Diabetic autonomic neuropathy further impairs myocardial ischemia signaling, degrading neural sensing systems innervating the heart (*65*).

Regardless of the mechanism, myocardial sensory networks cannot reliably detect myocardial ischemia. In this preclinical study, we address this deficiency of myocardial sensory networks using ANNs. ANNs have taken design cues from several systems over the years, ranging from the early days of parallel distributed processing to recent ANN architectures that mimic mammalian neural networks [reviews in (*66*, *67*)]. Future ANN-based approaches hold promise for mitigating numerous shortcomings of physiological systems. Aside from controlling a therapeutic device during disease, ANNs can now also assist healthy humans across an array of scenarios (*68*). These findings further highlight several areas of opportunity for ANNs to enhance human function, during either disease or even healthy states.

### Reversing spontaneous myocardial ischemia using a responsive closed-loop bioelectronic medicine

Silent myocardial ischemia can contribute to the vast majority of all ischemic episodes. High rates of silent myocardial ischemia lead to increases in myocardial injury, myocardial infarction, and sudden death (*6*, *8*, *69*, *70*). Treating silent and symptomatic myocardial ischemia can reduce rates of myocardial injury, myocardial infarction, and death (*5*–*8*). Even specifically treating patients that have significant symptomatic myocardial ischemia can affect pathophysiology during episodes of silent myocardial ischemia, due to its higher prevalence. Treating myocardial ischemia using a pharmacological medicine can promote vasodilation and/or reestablish an appropriate myocardial oxygen supply-demand ratio (*7*, *71*). VNS mimics these desired effects via cholinergic modulation, decreasing intracellular calcium, presynaptic inhibition of norepinephrine release, coronary vasodilation, and other mechanisms (*15*–*17*, *37*). Bioelectronic control of these physiological cascades motivated the use of VNS in this preclinical study.

Bioelectronic medicines are beginning to address several shortcomings of pharmacological medicines (*26*, *72*, *73*). Although pharmacological medicines can be effective, they do not target specific tissues, leading to side effects (which can ultimately limit pharmacological medicine adherence overall). Bioelectronic medicines can address this limitation, via stimulating specific nerves and therefore targeting specific tissues, for a more localized effect. Furthermore, several disease episodes are spontaneous and may only occur for several minutes per day. Bioelectronic medicines can be dynamically switched on and off as needed, unlike pharmacological medicines that are active for several hours a day. Importantly, bioelectronic medicines can provide on-demand benefit via closed-loop activation. This on-demand attribute of bioelectronic medicine can reduce desensitization of target receptors, further mitigate side effects, and ultimately improve therapeutic efficacy. Overall, bioelectronic medicines mitigate several shortcomings of pharmacological medicines, providing spatial and temporal specificity to improve therapeutic outcomes and reduce off-target effects.

Our findings extend previous studies that apply vagal modulation during myocardial ischemia (*74*–*76*) and specifically highlight the ability of responsive closed-loop VNS control (Fig. 5 and fig. S6). Notably, only responsive closed-loop VNS (with efferent vagal fibers intact) reversed the major physiological features of myocardial ischemia (Fig. 5 and fig. S6). Efferent cervical vagal fibers innervate both the atria and ventricles (*15*, *16*). Acetylcholine release from efferent vagal fibers can mitigate elevated chronotropy, inotropy, afterload, and myocardial oxygen consumption seen during myocardial ischemia (*15*–*17*, *74*–*76*). Our results demonstrate that closed-loop intact VNS decreases overall myocardial work, important for preventing cell death and injury during myocardial ischemia.

During myocardial ischemia alone, we observed depression of ECG segments during both systole and diastole (Fig. 3). These ECG epochs depress during the initial stages of myocardial ischemia, indicative of ischemic currents (*40*, *43*, *44*). Closed-loop intact VNS reduced chronotropy, afterload, myocardial oxygen consumption, and other factors leading to a full reversal of ST epoch depression (Fig. 5). This result importantly suggests a complete reversal of subendocardial ischemia (*40*) during closed-loop intact VNS. Overall, these findings support the hypothesis that closed-loop intact VNS suppresses ischemic currents, via a responsive increase in parasympathetic drive restoring myocardial oxygen balance.

Last, the cardiovascular effects of VNS required precise timing and delivery of stimulation during spontaneous ischemic episodes. Open-loop VNS was not programmed to respond during spontaneous myocardial ischemia and thus generally failed to affect physiological features of myocardial ischemia (Fig. 5 and fig. S6). Therefore, open-loop VNS may simply miss random myocardial ischemia events. We used an open-loop VNS paradigm representative of human cardiovascular VNS studies, nearing the upper limit of clinically tolerable VNS levels [20% duty cycle at 2 to 2.5 mA (Table 1) (*52*, *53*)]. From a translational perspective, the closed-loop VNS paradigm used here should deliver significantly less VNS compared to open-loop VNS over time. For example, several clinical studies indicate that myocardial ischemia can occur for several minutes and up to ~1 hour/day (*4*, *77*). Therefore, to responsively mitigate myocardial ischemia, closed-loop VNS may only be needed for ~1 hour a day or less. Over a 24-hour period, closed-loop VNS should also deliver ~1 to 2 orders of magnitude less VNS compared to open-loop VNS. The total charge delivery of VNS is important for future safety studies aiming to treat spontaneous myocardial ischemia with VNS. These results may motivate future studies to optimize the total amount of stimulation delivered using responsive bioelectronic medicines, keeping in mind the desired safety and efficacy.

### AI-enabled medicines: Opportunities and challenges

State-of-the-art machine learning methods provide powerful capabilities for pattern recognition that, in many cases, exceed the abilities of expert humans. The financial industry was an early adopter of neural network models for forecasting stock market index, energy demand, and real estate prices, prompted initially by a need to model nonlinear multivariate datasets. In medicine, the role of AI has been increasing steadily (*78*), especially in the field of radiology where AI-enabled systems are used not only for the detection and interpretation of images but also for scheduling and triage, clinical decision support systems, and several other critical steps of the radiology workflow.

The uptake of AI-based solutions is driven by their capacity to ingest and comprehend vast quantities of data, permitting a more comprehensive assessment of a patient’s condition. Included in this is the ability to detect dynamic features that are not apparent in the typical snapshot evaluations that are performed in the clinic (e.g., blood pressure and heart rate at a single time point) (*79*). We leverage these capabilities of AI systems to dynamically detect and correct pathological cardiovascular events in vivo (Fig. 5 and fig. S6), similar to previous studies using responsive therapies for cardiovascular treatment (*80*, *81*).

Despite the clear benefits of AI-enabled technology solutions, trustworthiness is a major barrier to the adoption of AI-based diagnostics and especially intervention. Some patients and physicians may be reluctant to allow a computer to make health care decisions. A recent survey of radiologists, information technology specialists, and industry representatives found that only 25% of the 123 people surveyed expressed confidence in results obtained by AI systems used in radiology, and the vast majority (~91%) emphasized the need to validate the algorithms used in these systems (*82*). Strategies for building trust include the creation of “explainable AI” that provides greater transparency and traceability, especially for systems that rely on deep learning architectures that are particularly opaque (*28*, *83*). Improved methods for data and model visualization may facilitate interpretability and explainability in medical AI systems (*27*, *29*) and were leveraged in the current study (via autoencoders, dimensionality reduction, and unsupervised clustering; Figs. 6 and 7). Building trust will likely be achieved gradually through an evolution of clinical trials that demonstrate with hard evidence the benefits of AI-based approaches in improving patient care.

### Translational research perspective

Similar to previous preclinical studies, our model of acute myocardial ischemia used high catecholamine tone to precipitously disrupt myocardial energetic imbalance and facilitate electrocardiographic dysfunction (Figs. 1 and 3). The model was motivated by several previous studies using high catecholamine tone to acutely induce myocardial ischemia, ST segment changes, and distributed cell death or transmural necrosis when the infusion is prolonged, even in healthy animals (*33*–*38*). The high catecholaminergic state generated by our model influences heart rate, myocardial contractility, and vascular tone (afterload), each of which is targeted by current pharmacologic therapies in treating patients with chronic ischemic heart disease and a high burden of myocardial ischemia (*7*, *71*). Furthermore, high catecholaminergic tone is of interest in gaining understanding of new clinically recognized complex cardiovascular disease states. Although catecholaminergic tone may be a final common pathway in cardiovascular disease, new disease attributes are emerging related to further understanding this set of complex cardiovascular processes.

Two attributes of interest include ischemia and no obstructive coronary artery disease (INOCA) and coronary microvascular dysfunction (CMD) (*84*). INOCA occurs without a significant stenosis of the coronary arteries (*84*). CMD involves structural and/or functional components, affecting the smaller-diameter coronary vessels (i.e., <~200-μm diameter vessels) (*84*). Excessive levels of catecholamines can adversely affect coronary microvascular function and facilitate myocardial energetic imbalance in the absence of obstructed coronary arteries (e.g., via significant coronary vasoconstriction, coronary vasospasm disrupting coronary blood flow, and/or calcium overload) (*33*–*38*). Therefore, our preclinical model may induce acute ischemia via correlates of CMD in animals without obstructed coronary arteries. Regardless, further mechanistic study is needed in models of INOCA and/or CMD, for example, quantifying the effects on coronary flow and perfusion.

Last, VNS is a Food and Drug Administration approved intervention for an array of disorders, has an excellent safety profile, and has promise for reactively reversing episodic cardiovascular dysfunction. Regardless, limitations of VNS should be kept in mind and assessed in future studies. For example, inflammation is a critical contributor to atherosclerosis during cardiovascular disease. Although closed-loop VNS may not immediately halt inflammation, VNS can play a large role in mitigating inflammation over longer time periods (e.g., via decreasing levels of several inflammatory cytokines) (*14*, *85*). Therefore, seemingly acute closed-loop VNS may mitigate cardiovascular inflammatory cascades related to atherosclerosis over longer periods of time. Furthermore, acute closed-loop VNS cannot immediately reverse a significant epicardial coronary stenosis. However, most of the coronary vascular resistance is due to the smaller coronary microvasculature. Reports importantly demonstrate that VNS can effectively dilate the critical coronary microvasculature, improving myocardial perfusion in preclinical animal models and humans (*37*, *86*). This can also subsequently enhance myocardial electrical stability and mitigate arrhythmias (*54*, *75*, *76*). We look forward to future studies assessing these factors for further bolstering the translational potential of closed-loop VNS for treating cardiovascular dysfunction.

## MATERIALS AND METHODS

### Overview

All procedures were approved by the Institutional Animal Care and Use Committee of QTest Labs (Columbus, OH). Adult male Sprague-Dawley rats (~400 to 750 g; *n* = 14) used in this study were housed one per cage (12-hour light/dark cycle; ad libitum access to food and water). The general aims of the study were as follows: (i) to establish a model of myocardial ischemia, (ii) to use machine learning approaches to decode cardiovascular state changes, (iii) to determine whether responsive closed-loop VNS controlled by an ANN can significantly mitigate spontaneous myocardial ischemia, and, last, (iv) to assess machine learning architectures for enhancing interpretability and facilitate detection of new emerging cardiovascular states. To acquire cardiovascular data, we recorded a lead II ECG, arterial blood pressure (aBP), and a photoplethysmogram (PPG) (schematic of experimental interfaces in fig. S1A). Analyses were performed in either MATLAB or Python.

### Surgery and interface placement

Animals were first administered carprofen (5 mg/kg, subcutaneous injection) and initially anesthetized using isoflurane, similar to previous studies assessing the effects of VNS on cardiovascular physiology (*87*, *88*). Isoflurane was administered via a tracheotomy interface for the duration of the experiment (1.3 to 1.7 vol. %; fig. S1A, light red tube). Animals were supine throughout the procedure. Core body temperature was maintained at ~37°C using a heating platform placed under the animal (Vestavia Scientific; Birmingham, AL).

The following six interfaces were next placed (schematic in fig. S1A): Catheters were placed within the (i) right and (ii) left femoral veins for intravenous administration of dobutamine and/or norepinephrine (see the “Inducing myocardial ischemia via catecholamine infusion” section for more details on agent administration); (iii) aBP was recorded within the right carotid artery using a solid-state blood pressure catheter (2 French; SPR-407 Mikro-Tip; Millar, Houston, TX) and sent to a blood pressure amplifier (DA100C; BIOPAC, Goleta, CA); (iv) a lead II ECG was recorded using three hydrogel electrode contacts (ground: left arm, V+: right arm, V−: left leg) connected to an ECG amplifier (ECG100C; BIOPAC, Goleta, CA); (v) blood oxygen saturation level (SpO_2_) was recorded from the right hindpaw (OXY200; BIOPAC, Goleta, CA); and (vi) the left cervical vagus nerve was interfaced with a bipolar platinum iridium cuff electrode for delivering VNS, similar to our previous studies (*24*–*26*). The bipolar VNS cuff electrode was tethered to a digitally controlled stimulator (Digitimer DS5; Hertfordshire, UK). All instruments and stimulators were robustly electrically isolated to prevent stimulation artifact during cardiovascular data recordings.

### VNS cuff implant

We interfaced with the left cervical vagus nerve to enable cardiovascular control, similar to several previous preclinical (*17*, *87*–*89*) and human studies (*52*, *90*). VNS was delivered with the following stimulation parameters: biphasic square wave, 2 to 2.5 mA, 300-μs pulse width, and 30 Hz. VNS was delivered during closed-loop or open-loop stimulation modes (see the “Modes of VNS delivery” section for more details). These VNS parameters are similar to previous preclinical studies using VNS for cardiovascular control (*17*, *87*–*89*) and fall within clinically relevant stimulation ranges used in VNS clinical trials for cardiovascular therapy [Table 1 (*52*, *53*)].

### System control for recording, stimulation, and infusion

A schematic of the experiment and interfaces is shown in fig. S1A. All data were collected using a National Instruments USB-6259 data acquisition system (DAQ). The DAQ was controlled using MATLAB 2019a via a custom graphical user interface (MathWorks; Natick, MA). We recorded five signals during the experiments: (i) the voltage sent to the VNS cuff electrodes, (ii) the current drawn from the VNS cuff electrodes, (iii) the lead II ECG waveform, (iv) the aBP waveform, and (v) the SpO_2_ signal. Signals #1 and #2 were only active during VNS events. We also enabled three outputs during the experiments, as needed: (i and ii) triggers controlling the two catecholamine infusion pumps (KDS-200; Kent Scientific, Holliston, MA) and (iii) a trigger for the module controlling VNS. The DAQ operated overall at 10 kHz. This rate was needed to create VNS trains with the appropriate waveform morphology (e.g., biphasic square waves with the appropriate shape and resolution). Recorded signals were down-sampled and conditioned online as needed.

### Inducing myocardial ischemia via catecholamine infusion

Cardiovascular stress and myocardial ischemia were induced using intravenous infusion of dobutamine (~2 to 3 μg kg^−1^ min^−1^) and/or norepinephrine (~2 to 3 μg kg^−1^ min^−1^). Pilot studies were performed to assess dose-dependent effects. These agents and dose rates have been used in several previous studies (*33*, *35*, *37*, *38*, *91*).

### Online cardiovascular signal conditioning and feature extraction

A schematic of feature extraction wave points is shown in Fig. 2. The subcomponents of features were first identified online via the following signal conditioning processes (occurring every 100 ms): (i) A 10-kHz sampled epoch of the ECG and aBP waveforms were first down-sampled to 500-Hz sampled waveforms; (ii) for the ECG epoch (Fig. 2A), the R waves were first detected using the “Peak Prominence” attribute of the “findpeaks” function in MATLAB 2019a. Window-based detection was then used to identify the P, Ta, S, and T wave correlates (Fig. 2A). The time and voltage level of the given ECG wave correlates were recorded; (iii) for the given aBP epoch (Fig. 2B), the systolic and diastolic pressure wave points were detected using the “Peak Prominence” attribute of the “findpeaks” function in MATLAB 2019a. The mmHg values of the systolic and diastolic aBP levels were recorded; (iv) inhalation and exhalation cycles were encoded into the low-frequency components of the aBP waveform. The linear envelope of the aBP waveform was calculated to extract the respiratory cycle time series. Inhalation points (i.e., breaths) were next detected and recorded using the “Peak Prominence” attribute of the “findpeaks” function in MATLAB 2019a.

The 13-element feature vector was lastly constructed from the above ECG and aBP waveform attributes via the following calculations (again, occurring every 100 ms):

1) Feature #1: ST epoch level (millivolts) = voltage level of the S wave + voltage level of the TP interval

2) Feature #2: Heart rate [beats per minute (bpm)] = RR interval (seconds)/60 s

3) Feature #3: QRS duration (milliseconds) = relative Q wave to S wave duration

4) Feature #4: RT duration (milliseconds) = R wave to T wave duration

5) Feature #5: ST duration (milliseconds) = S wave to T wave duration

6) Feature #6: Ta level (millivolts) = voltage level of the Ta wave + voltage level of the TP interval

7) Feature #7: R level (millivolts) = voltage level of the R wave + voltage level of the TP interval

8) Feature #8: ST slope (millivolts per second) = [T wave level (millivolts) − S wave level (millivolts)]/[T wave time (seconds) − S wave time (seconds)]

9) Feature #9: Diastolic pressure (mmHg) = minimum pressure level during diastole

10) Feature #10: Systolic pressure (mmHg) = maximum pressure level during systole

11) Feature #11: MAP (mmHg) = [systolic pressure (mmHg) + diastolic pressure (mmHg)]/2

12) Feature #12: Pulse pressure (mmHg) = systolic pressure (mmHg) − diastolic pressure (mmHg)

13) Feature #13: Breath rate [breath rate per minute (brpm)] = breath count/60 s

This feature vector contains relatively simple features that facilitate decoding interpretation and can be extracted for decoding without the need for burdensome compute power. To smooth the data, the feature vector was averaged over a 4-s sliding window and was updated every 100 ms during real-time recordings. The feature data were recorded for offline analysis and were also used for online decoding in vivo (see the “Decoding myocardial ischemia using an ANN” section for more details). Features from a subset of recordings were independently validated using emka ecgAUTO software for reference (emka; Sterling, VA).

### Decoding myocardial ischemia using an ANN

#### Overview

A schematic of the ANN architecture and decoder outputs is shown in fig. S3 (A and B). We used an ANN architecture and a supervised learning approach to decode four different cardiovascular states (i.e., classes): (i) rest (i.e., no drug infused), (ii) dobutamine infusion (D), (iii) norepinephrine infusion (NE), and (iv) a combined dobutamine and norepinephrine infusion (D + NE). The decoder outputs were assessed both offline and online to evaluate performance. Online predictions were used to either validate the ANN model or control closed-loop VNS.

#### Recording events and data labels

Each recording contained the following events: (i) time 0 s, start of initial data streaming; (ii) time 4 s, initiation of 4-s sliding window used for averaging features (sliding window increment per observation, 100 ms); (iii) time 34 s, initiation of decoding (allows for 30 s of background feature data; these background feature data are used to both baseline-subtract and standardize the subsequent recorded feature data); (iv) time 94 s, start of a given infusion; (v) time 214 s, end of infusion, decoding, and recording. To determine data labeling time points for supervised learning and ANN architecture attributes, we initially recorded pilot data from *n* = 5 animals. On average, all 13 features statistically changed from baseline levels ~15 s after infusions were started (change averaged across all three infusion types). Said differently, average physiological changes across infusion types occurred at 109 s. Therefore, the “rest” class label occurred from 4 to 109 s, and the given infusion’s class label occurred during the infusion period from 109 to 214 s. This labeling approach enabled both physiologically motivated data labels and balanced durations of rest and a given cardiovascular state during a given recording (for mitigating class imbalance).

#### Grid search for ANN architecture and hyperparameters

We used a grid search to determine the ANN architecture and hyperparameters (schematic of the final ANN architecture in fig. S3A). The grid search leveraged the same pilot data that were used for creating the data labels described above (again from *n* = 5 animals; a total of data ~1.2 million points) and was performed on a computer with a graphical processing unit (NVIDIA GeForce GTX 1080; Santa Clara, CA). Overall accuracy (i.e., average accuracy across all four classes) was used as the given algorithm’s performance metric. Our preliminary analysis demonstrated the best performance using two hidden layers (a dense layer followed by an LSTM layer).

We next assessed combinations of the following architecture and hyperparameter values: (i) number of units in the dense layer (100, 250, or 500), (ii) number of units in the LSTM layer (100, 250, or 500), (iii) drop-out layer mask (between both the dense and LSTM and the LSTM and output layers; at 25, 50, or 75%), (iv) mini-batch size (25, 50, or 75% of total data), and (vi) early stopping criteria (reaching either 95 or 98% overall accuracy during training). The following were fixed during the grid search: sequence input layer size (13 units), output layer size (4 units), optimization algorithm (Adam), gradient decay metric (0.8), learning rate (0.01), gradient threshold (2), and L2 regularization metric (0.0005). The following ANN architecture and hyperparameters consistently performed the best and were used throughout the study: architecture = sequence input layer (13 units), dense layer (250 units), drop-out layer mask (50%), LSTM layer (100 units), drop-out layer mask (25%), and output layer (4 units); hyperparameters = mini-batch size (75%) and early stopping (reaching 98% overall accuracy during training).

#### Algorithm performance evaluation

Online in vivo assessments (related to Fig. 5 and fig. S5): We performed online in vivo decoding of cardiovascular state and ANN-controlled VNS in a total of *n* = 9 animals. Overall, we modeled a clinical use case for the ANN. We continuously added to the base training set across experiments and performed supervised updating of the ANN within a given animal for subsequent real-time prediction and closed-loop ANN-VNS control (this paradigm, in part, mimics a use case involving the continuous addition of new recorded data and subsequent model updating across time). Experimental design details are as follows: (i) The initial training set consisted of the pilot data; (ii) each subsequent new animal then contributed six more recordings to the base training set (infusion order randomized; two infusions of D, two infusions of NE, and two infusions of D + NE); (iii) for a given new animal, a new ANN model was updated/retrained and validated online in vivo; (iv) online testing consisted of real-time prediction during three infusions of the target ischemic state (D + NE). We report overall accuracy for online in vivo ANN performance (fig. S5A).

Offline assessments (related to Fig. 4 and figs. S3 and S4): We also assessed algorithm performance offline [pilot data (*n* = 5) + experimental data (*n* = 9)]. A 10-fold cross validation was used to appraise the performance of the ANN and other types of classifiers for comparison (using 80%/20% train-test splits, respectively). We compared ANN performance to three other classifier types: (i) an ANN-NO-LSTM architecture (via replacing the LSTM layer with a second hidden dense layer of the same size); (ii) an SVM; and (iii) an LDA. Similar to the ANN, we performed a hyperparameter grid search to optimize the performance of both the SVM and LDA. We report overall accuracy for all classification approaches (fig. S3C).

### Modes of VNS delivery (related to Fig. 5 and figs. S5 and S6)

We assessed the effects of ANN-VNS during episodes of spontaneous myocardial ischemia (i.e., “Isch.” induced by D + NE). Figure S5B shows the average ANN decoder outputs during real-time predictions in vivo (decoder outputs averaged across all *n* = 9 animals). Closed-loop VNS was triggered when the Isch. decoder output score was greater than 0.5, representing a class probability greater than 50% [similar to our previous decoding and device activation assessments in (*92*)]. Once triggered by the ANN, VNS remained active for the remainder of the infusion (i.e., up to 214 s). All instrumentation and stimulators were robustly isolated to prevent stimulation artifact during recordings.

#### Additional VNS controls

We performed two VNS controls, complementing ANN-VNS. The first VNS control condition was open-loop VNS (data presented in Fig. 5 and fig. S6B). We used a 20% VNS ON/80% VNS OFF duty cycle for the open-loop VNS condition to model the preprogrammed open-loop VNS duty cycles used in clinical trials for cardiovascular treatments [Table 1 (*52*, *53*)]. Open-loop VNS recordings lasted for a total of ~500 s, with two to three recording replicates within an animal (across *n* = 5 animals). A D + NE infusion was started at a randomized time during an open-loop VNS recording, using the same 2-min infusion duration (to model the spontaneous nature of ischemia, relative to a preprogrammed VNS paradigm).

The second VNS control condition was ANN-VNS following a vagotomy caudal to the cervical VNS site (data presented in fig. S6C). The left cervical vagus nerve (where the VNS cuff was implanted) was cut using surgical scissors, and the cut ends were further separated by ~1 mm to ensure a complete vagotomy. Recording and VNS were resumed approximately 30 min after the vagotomy to allow for physiological equilibration. We performed three recording replicates within an animal (across *n* = 3 animals).

### Data analyses for assessing cardiovascular feature changes (with or without VNS)

Several cardiovascular features shown throughout the manuscript are presented as a change from baseline (i.e., ∆ relative to baseline). For the given feature, baseline activity from the first 30 s of a recording was used to create the baseline-subtracted feature time series (see the “Online cardiovascular signal conditioning and feature extraction” section for details on feature creation). A given feature was further processed as follows to assess effects:

1) Related to Fig. 1B: RPP across time was not a component of the overall 13-element feature vector but was calculated offline, similar to previous studies (*39*): RPP = (heart rate × systolic blood pressure)/100.

2) Related to Fig. 1D: QTc across time was not a component of the overall 13-element feature vector but was calculated offline using the emka ecgAUTO software and Bazett’s formula [similar to previous studies (*41*, *42*)]: QTc = (QT interval; milliseconds)/√ (RR interval; milliseconds).

3) Related to Fig. 1 (F and G): We observed three types of arrhythmias during the induction of cardiovascular stress and acute myocardial ischemia: VT, PAC, and PVC. We report the overall arrhythmia incidence across infusion types.

4) Related to Fig. 3: A given feature’s ∆ value was its average during the entire infusion period, relative to baseline.

5) Related to fig. S2 (A and B): We compared the variability (i.e., entropy) of cardiovascular changes for our preclinical rat data and human data collected in other studies. We used the following human cardiovascular data acquired from the physionet.org database (*46*): fig. S2A, “human 1,” recorded in the intensive care unit (*47*); fig. S2B, “human 2” (*48*) and “human 3” (*49*), recorded during ambulatory episodes of myocardial ischemia. The cardiovascular feature time series were next prepared using either all 13 features (fig. S2A) or only the 8 ECG features (fig. S2B). The feature time series for the human data used a modified version of the rat feature extraction algorithm. Entropy was lastly calculated and reported using the “entropy” function in MATLAB 2019a.

6) Related to fig. S2C: We assessed the relationship between different pairs of cardiovascular states and report the Pearson correlation coefficient *R*. Within each animal (*n* = 9), we calculate the *R* values for all possible pairs of infusions using the feature time series (e.g., a D + NE versus NE correlation). We plot each animal’s average *R* value (single points in the figure) across the three different types of infusion correlations.

7) Related to fig. S5C: We assessed the relationship between the Isch. (D + NE) decoder output and the D + NE ST epoch level (following min-max normalization to allow for comparison). We report the Pearson correlation coefficient *R*.

8) Related to Fig. 5 (C and D) and fig. S6: In Fig. 5D, we report an additional feature: the QT/TQ ratio [related to arrhythmia probability (*54*); calculated using the emka ecgAUTO software]. Overall, for Isch., a given feature’s ∆ value was its average during the entire infusion period, relative to baseline. Overall, for “Isch. + ANN-VNS,” “Isch. + OL-VNS,” and “Isch. + ANN-VNS + (xcv),” a given feature’s ∆ value was its average while VNS was on, relative to baseline.

### Detecting new emerging cardiovascular stress states (related to Fig. 6 and fig. S7)

In a subset of animals (*n* = 4), we recorded the 13 features during infusions at a much higher dose rate (10 μg kg^−1^ min^−1^) across three infusion types: “high-dose dobutamine,” H-D; “high-dose norepinephrine,” H-NE; “high-dose dobutamine and norepinephrine combined,” H-D + NE. These recordings at a ~5× higher dose rate presented a notably different feature profile during a given infusion and were used for subsequent emerging stress state detection analyses. We appraised the ability of three techniques to detect these emerging stress states [emerging state/outlier detection technique review (*55*)]: (i) an LSTM-AE, (ii) a Sparse-AE, and (iii) an Iso-Forest. Each technique was optimized using a grid search on a subset of the data [LSTM-AE major parameters: encoder layer (input) = 2730 units, hidden layer = 256 units, decoder layer (output) = 2730 units, and L2 regularization = 0; Sparse-AE major parameters: encoder layer (input) = 546 units, hidden layer = 50 units, decoder layer (output) = 546 units, L2 regularization = 0.1, and sparsity proportion = 1; Iso-Forest major parameters: n estimators = 2, max features = 140, and outlier proportion = 0.16]. We lastly performed a 10-fold cross validation to appraise the performance of the three techniques (using 80%/20% train-test splits, respectively). We report “emerging stress state detection sensitivity” for the three techniques (i.e., true-positive rate when presented with a new emerging stress state, averaged across the three types of emerging stress states; Fig. 6).

### Visualizing the cardiovascular latent space using LSTM-AEs and UMAP (related to Fig. 7, A and B, movie S1, and fig. S8)

We used the hidden layer of the LSTM-AE combined with the dimensionality reduction method UMAP (*56*) to visualize interpretable two-dimensional representations of the emerging stress states (i.e., the cardiovascular latent space). The hidden layer of autoencoders and UMAP can readily be used for generating latent features and dimensionality reduction (*57*). The hidden layer of the LSTM-AE (256 elements) was labeled and passed into the UMAP algorithm for supervised dimensionality reduction (schematic of architecture in Fig. 7A; final UMAP hyperparameters: n_neighbors = 15; min_dist = 0.1; n_components = 2). The ability to separate the two-dimensional known and unknown emerging stress state points in the cardiovascular latent space was assessed using a simple linear SVM (related to fig. S8).

### Identifying new emerging cardiovascular stress state types using unsupervised clustering (related to Fig. 7C)

We performed unsupervised clustering of the two-dimensional cardiovascular latent space points using the HDBSCAN method. HDBSCAN is a robust unsupervised clustering technique that deals well with diverse clustering scenarios (*58*). For a given stress state presentation scenario (combinations of one, two, or three emerging stress types), the unsupervised HDBSCAN method was first challenged to cluster the two-dimensional cardiovascular latent space points (i.e., determine the number of emerging stress states present). We next calculated clustering performance [V-measure × 100% (*59*); V-measure is a well-studied metric for assessing clustering quality; 0, completely random clustering and 1, perfect clustering]. In this implementation, V-measure–based performance also quantifies correlates of several processes including the LSTM-AE’s ability to generate useful latent space vectors, the quality of the subsequent UMAP dimensionality reduction, and the quality of the final HDBSCAN method. We calculate and report performance (Fig. 7C) averaged across the seven stress state presentation scenarios (H-D alone; H-NE alone; H-D + NE alone; H-D and H-NE; H-D and H-D + NE; H-NE and H-D + NE; and H-D, H-NE, and H-D + NE).

### Statistics

Normality tests were performed for each analysis to determine whether parametric or nonparametric statistics should be used. All statistical tests were two-tailed unless otherwise noted and were performed in GraphPad Prism. An alpha level of 0.05 was accepted for significance, unless Bonferroni corrections are noted. Chance performance levels were generated by randomly permuting the true data labels 10 times, similar to previous studies (*92*, *93*).

We report the Pearson correlation coefficient *R* in the text for data from Fig. 1 and in fig. S2C. Differences in arrhythmia incidence were evaluated using a one-way ANOVA (related to Fig. 1G). The factor was infusion type with three levels: D, NE, and D + NE. Tukey’s post hoc test was used to determine differences across infusion types. Effects of infusions on the 13-element feature vector were evaluated using a separate one-way ANOVA for each feature (related to Fig. 3). The factor was infusion type with three levels: D, NE, and D + NE. Tukey’s post hoc test was used to determine differences for a given feature across infusion types.

Differences in classification performance were evaluated using a one-way ANOVA (related to fig. S3C). The factor was classifier type with four levels: ANN, ANN-NO-LSTM, SVM, and LDA. Tukey’s post hoc test was used to determine differences in performance across classifier types. A one-tailed independent-samples *t* test was used to determine whether ANN performance values were above chance levels (related to Fig. 4D, confusion matrix). A Bonferroni-corrected alpha value of 0.003 was used for significance (0.05/16 comparisons).

Differences in cardiovascular features were assessed using separate one-way ANOVAs for each main effect (related to Fig. 5C) and side effect (related to Fig. 5D). The factor was state type with three levels: Isch., Isch. + ANN-VNS, and Isch. + OL-VNS. Tukey’s post hoc test was used to determine differences across state types. VNS ON time was also assessed using a one-way ANOVA (related to fig. S5D).

Two-dimensional known and emerging stress state points in the cardiovascular latent space were separated using a simple linear boundary (related to fig. S8), and differences between actual and chance performance were assessed using a *t* test. Differences in emerging stress state detection performance were evaluated using a one-way ANOVA (related to Fig. 6). The factor was detection technique with three levels: LSTM-AE, Sparse-AE, and Iso-Forest. Tukey’s post hoc test was used to determine differences in performance across detection techniques. A receiver operating characteristic curve was generated for the LSTM-AE approach to present the raw mean squared error (MSE) data (related to fig. S7). Last, we report the performance for unsupervised clustering and detection of different types of emerging stress states (related to Fig. 7C; performance = V-measure × 100%) (*59*). We assessed differences between actual and chance performance using a *t* test [either averaged across the emerging stress state presentation scenarios (Fig. 7C) or assessed separately across the emerging stress state presentation scenarios (fig. S9)].

## Notes

View/request a protocol for this paper from *Bio-protocol*.
