# Supplementary material for: Dynamic detection and reversal of myocardial ischemia using an artificially intelligent bioelectronic medicine
Source: Sci Adv. 2022 Jan 5;8(1):eabj5473. doi: 10.1126/sciadv.abj5473 (PMC8730601; doi:10.1126/sciadv.abj5473)
Supplement: Supplementary file 1 — Figs. S1 to S9 Legend for movie S1 [file sciadv.abj5473_sm.pdf]

Science Advances
AAAS

Supplementary Materials for

Dynamic detection and reversal of myocardial ischemia using an artificially intelligent bioelectronic medicine

Patrick D. Ganzer*, Masoud S. Loeian, Steve R. Roof, Bunyen Teng, Luan Lin, David A. Friedenberg, Ian W. Baumgart, Eric C. Meyers, Keum S. Chun, Adam Rich, Allison L. Tsao, William W. Muir, Doug J. Weber, Robert L. Hamlin

*Corresponding author. Email: pxg487@miami.edu

Published 5 January 2022, *Sci. Adv.* **8**, eabj5473 (2022)
DOI: 10.1126/sciadv.abj5473

The PDF file includes:

Figs. S1 to S9
Legend for movie S1

Other Supplementary Material for this manuscript includes the following:

Movie S1

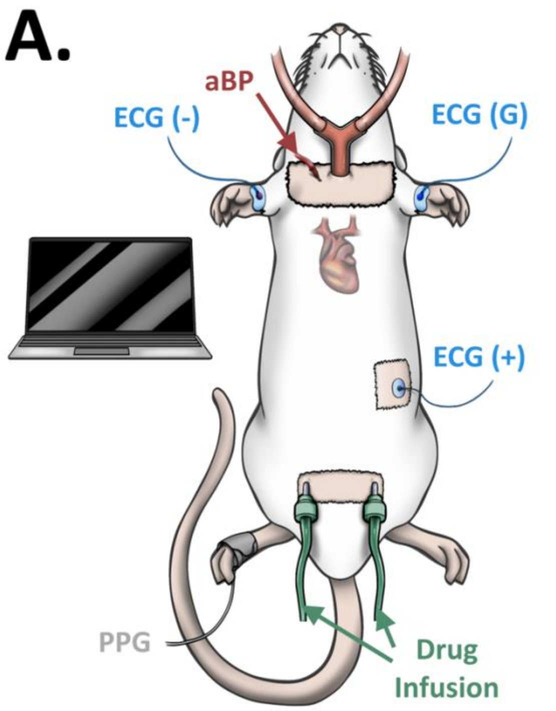

<u>Supplemental Figure S1. Cartoon Schematic of Experiment (related to Fig. 1, 2, & 3).</u> A. Cartoon schematic of the *in vivo* experiment and interfaces. All experiments were performed in isoflurane anesthetized rats (using tracheotomy, light red tube). We recorded arterial blood pressure from within the right carotid artery (aBP, red), a lead II electrocardiogram (ECG, blue patches; negative, positive, and ground electrodes noted), and a photoplethysmogram (PPG, right foot, black patch) during infusions of cardiovascular stress and myocardial ischemia inducing catecholamines into the femoral veins (catheters, green). All modules were synchronized and controlled electronically (laptop computer).

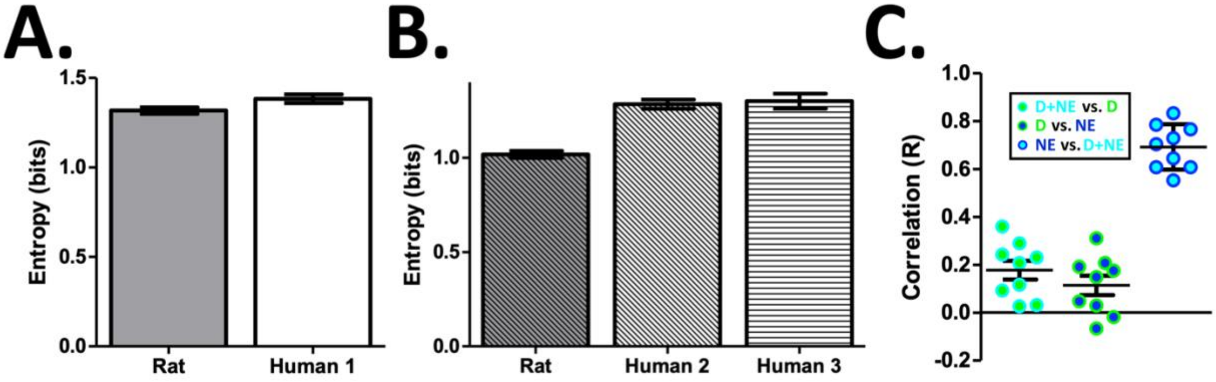

<u>Supplemental Figure S2. Cardiovascular Feature Data Exhibit Variability and State Overlap, Similar to Human Stress and Myocardial Ischemia (related to Fig. 3).</u> A & B. Cardiovascular feature data recorded from the rat ('Rat') demonstrated similar levels of variability (i.e., entropy) compared to cardiovascular feature data recorded from human subjects in either the intensive care unit (**A**; including all 13 features, 'Human 1') or from human subjects during ambulatory myocardial ischemia (**B**; including only ECG features, #1 - #8, 'Human 2' and 'Human 3'). **C.** Cardiovascular feature data from the rat also exhibited significant state overlap, specifically between NE and D+NE. These results support the hypothesis that cardiovascular stress and myocardial ischemia induced by D, NE, and D+NE infusions induce variability and state overlap in the cardiovascular data, similar to human cardiovascular stress states. Data presented are mean ± SEM.

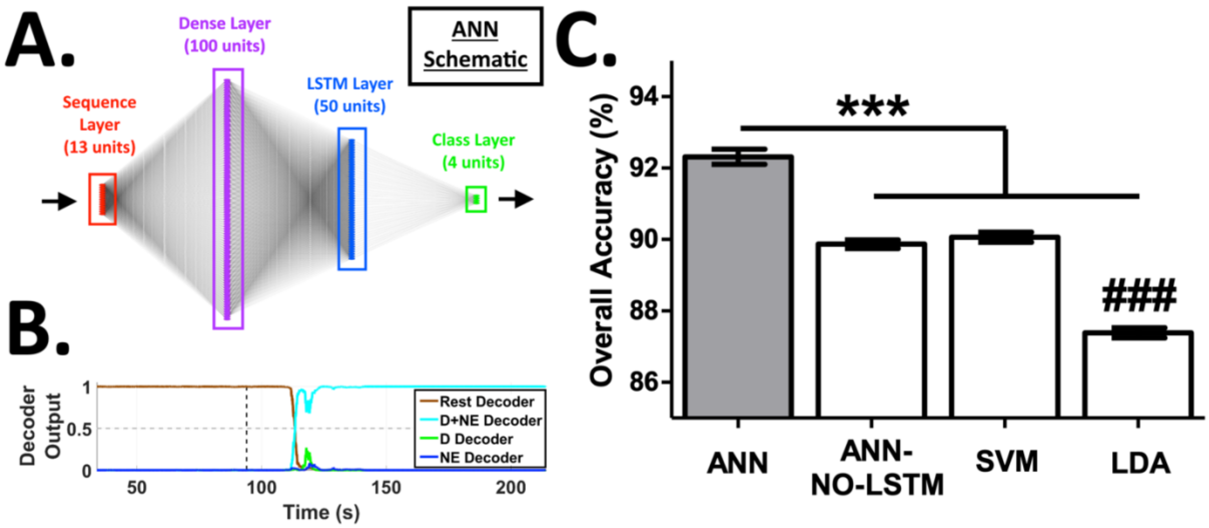

<u>Supplemental Figure S3. Artificial Neural Network (ANN) Architecture, ANN Decoder Outputs, and Superior Performance Compared to Other Classifiers (related to Fig. 4).</u> A. Cartoon schematic of the 4-layer ANN architecture (red: sequence input layer, 13 units; purple: dense layer, 100 units; blue: LSTM layer, 50 units; green: class output layer, 4 units). Please see the *Decoding Myocardial Ischemia Using an Artificial Neural Network (ANN)* section of the methods for more details on the ANN. **B.** A given recording begins with a 90 s period of rest (i.e., no agent infused) followed by a 120 s period of the given infused agent. Feature creation and decoding begins at 34 seconds to allow for the recording of sufficient baseline activity. Example ANN decoder outputs are shown across the 4 classes during an infusion of D+NE (a respective decoder output ranges from zero [low confidence in the respective class] to 1 [high confidence in the respective class]; gray dashed line = decoder significance threshold; black dashed line = infusion start). **C.** The ANN outperformed an artificial neural network without an LSTM layer (ANN-NO-LSTM), a support vector machine (SVM), and a linear discriminant analysis (LDA) (*** different at $p<0.001$; ### different from ANN-NO-LSTM or SVM at $p<0.001$). These results demonstrate the superior performance of ANNs and the importance of leveraging time series dependencies for cardiovascular state decoding. Data presented are mean ± SEM.

A.

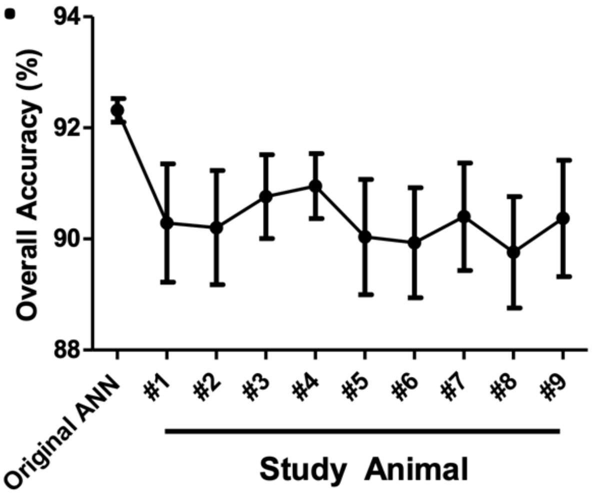

<u>Supplemental Figure S4. Fixed ANNs Demonstrate Significant Robustness and Generalization Out of Sample Across Time and Animals (related to Fig. 4).</u> **A.** Fixed ANNs were created to assess model generalization well out of sample to animals across the entire study. The original ANN performance level is shown as a reference (left, 'Original ANN'), where the model was trained on data from the entire study and therefore all animals. The remaining performance levels are shown for separate 'fixed' ANNs. A fixed ANN was only trained on the much smaller base pilot data set plus the given animal's data (noted by the study animal number). The given fixed ANN was then challenged to predict on the remaining animals in the study (several weeks into the past or future), without any model updating. Although there was a decrease in accuracy and an increase in prediction variance, fixed ANNs still generalized well across time and even to other animals (chance level of prediction = ~25%). These results indicate that ANNs used in the study are robust and can generalize well out of sample. Data presented are mean ± SEM.

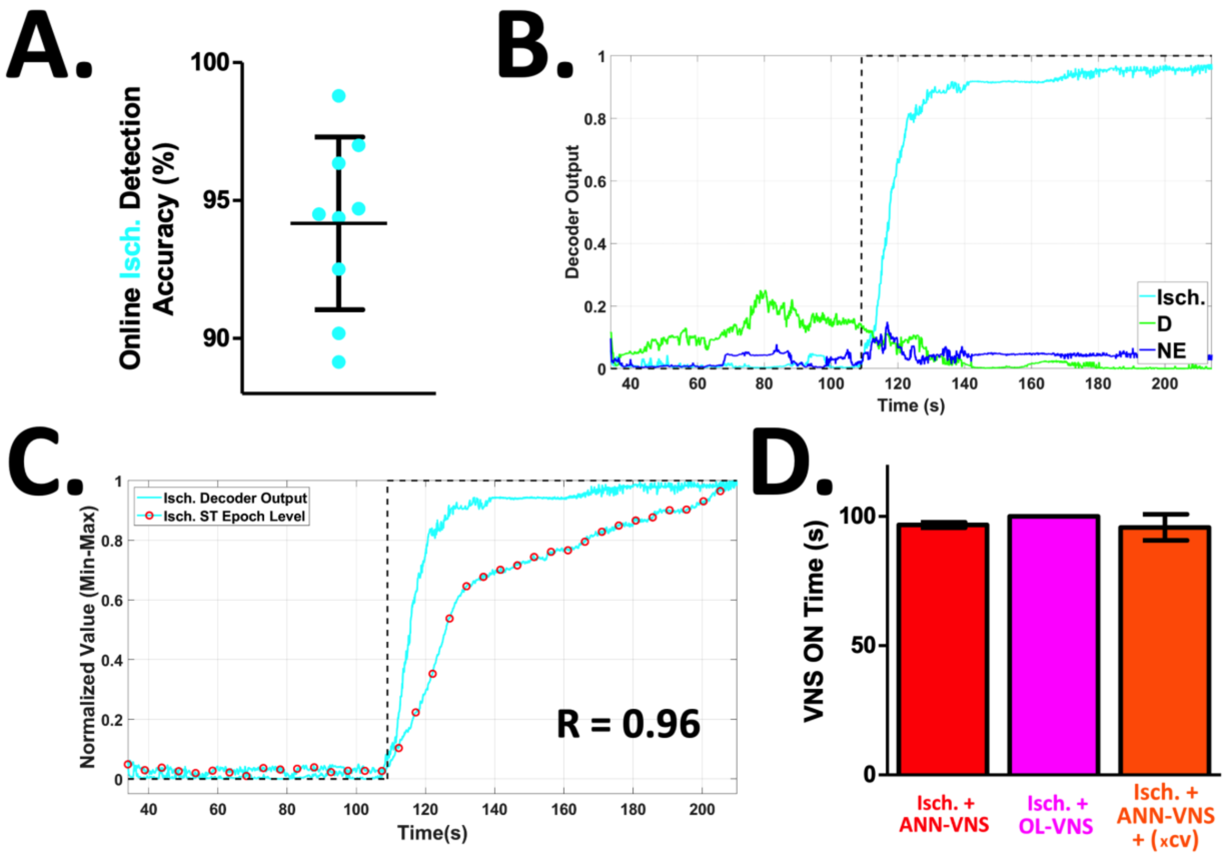

<u>Supplemental Figure S5. *In vivo* ANN Decoding Performance, Average ANN Decoder Outputs, and Amount of VNS Across Groups (related to Fig. 5).</u> **A.** The ANN performed *in vivo* online decoding of the target ischemic state (i.e., a D+NE infusion) with an overall accuracy of ~94% (cyan points = overall accuracies from single animals). **B.** ANN decoder outputs for the 3 cardiovascular stress states averaged across all animals from the *in vivo* experiments (N=9; black dashed line = labeled period for the given infusion). **C.** Isch. (D+NE) decoder outputs were highly correlated to ST epoch level changes ($R = 0.96$, $p<0.001$; data are min-max normalized to allow for comparison). **D.** All 3 VNS groups received the same amount of VNS (red = Isch. + ANN-VNS; magenta = Isch. + OL-VNS; orange = Isch. + ANN-VNS + (xcv)). Data presented are mean ± SEM.

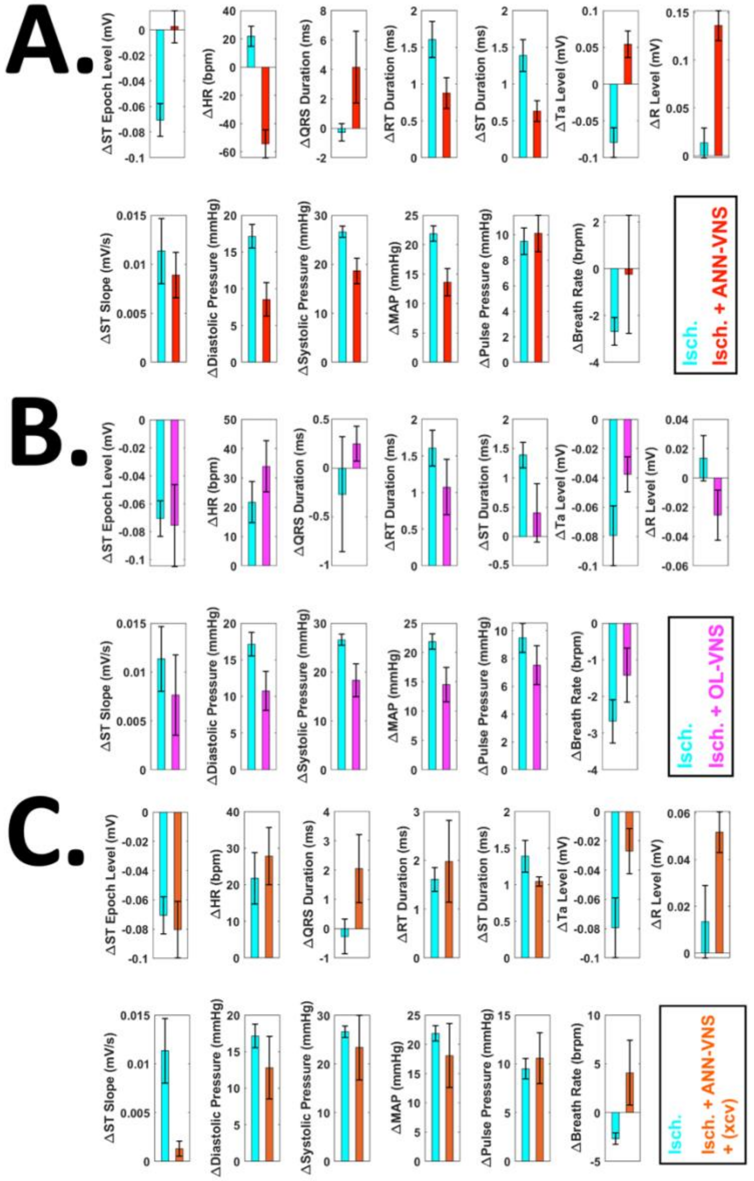

<u>Supplemental Figure S6. Effects Across All 13 Features Using Either Closed-loop VNS, Open-loop VNS, or Closed-loop VNS Following a Vagotomy Caudal to the VNS site (related to Fig. 5).</u> **A.** All 13 features during either D+NE ischemia alone (cyan, Isch.) or D+NE ischemia & closed-loop ANN-VNS (red, Isch. + ANN-VNS). We performed 2 controls to appraise the mechanism of ANN-VNS. Both preprogrammed open-loop VNS (all 13 features: **B**; magenta, Isch. + OL-VNS) and ANN controlled VNS following a vagotomy caudal to the VNS site (all 13 features: **C**; orange, Isch. + ANN-VNS + (xcv)) essentially failed to significantly affect cardiovascular pathophysiology induced by ischemia alone (cyan, Isch.). The results highlight the importance of both closed-loop VNS and vagal fibers engaged for reversing myocardial ischemia pathophysiology. Data presented are mean ± SEM.

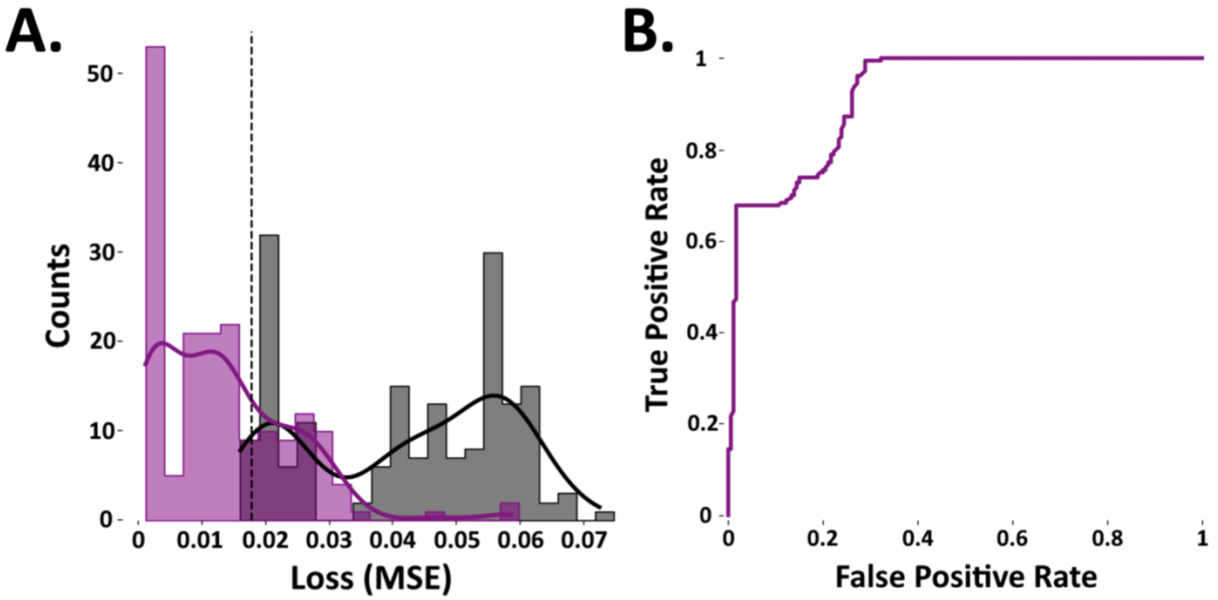

<u>Supplemental Figure S7. Emerging Cardiovascular Stress State Detection: Raw Mean Square Error (MSE) Reconstruction Distributions for The LSTM Autoencoder (LSTM-AE) (related to Fig. 6).</u> Using the LSTM-AE, emerging stress states were detected using a simple threshold method related to the reconstruction loss (i.e., mean square error or MSE), similar to previous studies (*55*). A high reconstruction loss is indicative of a new emerging state that has never been seen by the LSTM-AE, and a low reconstruction loss indicates that the state is generally known. The MSE loss distributions across all folds are shown for the LSTM-AE models for reconstruction of known stress states (**A**, purple; i.e., D, NE, and D+NE), or new emerging stress states (**A**, gray; i.e., H-D, H-NE, and H-D+NE). Colored curves (gaussian kernel fits) are shown on top of a given distribution (vertical dashed line = MSE threshold for determining known and new emerging stress states, optimized for accuracy). **B.** Receiver operating characteristic curve for the LSTM-AE technique, where true positive and false positive rates are plotted across a range of MSE thresholds.

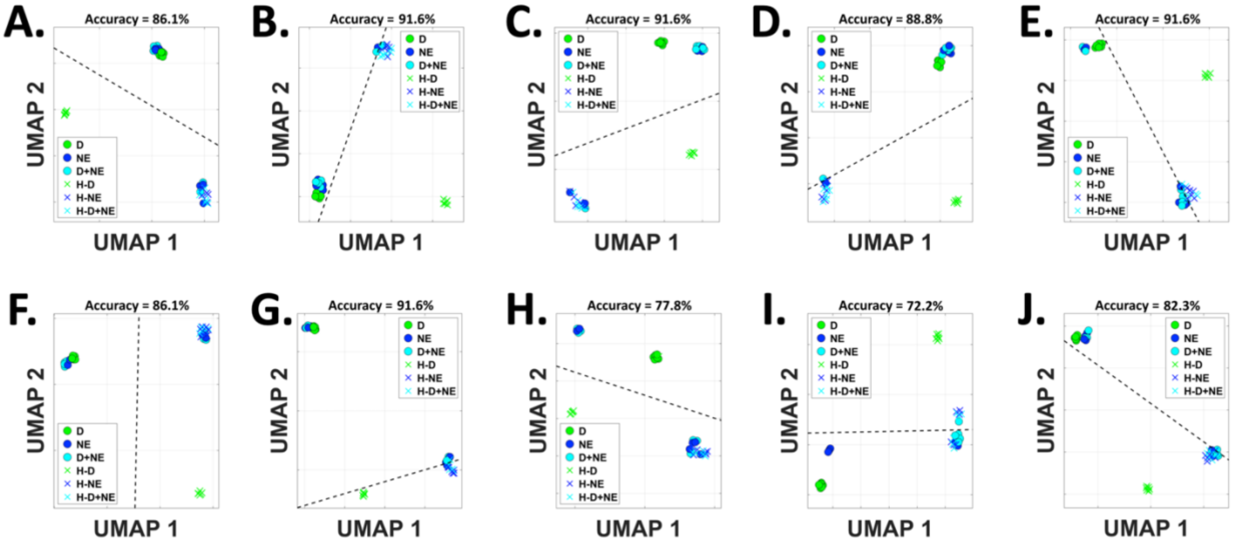

<u>Supplemental Figure S8. Known and Unknown Cardiovascular Stress States Within the ‘Cardiovascular Latent Space’: Raw Data from All Folds (related to Fig. 7B).</u> We assessed 2-dimensional representations of all known and unknown stress states within the ‘cardiovascular latent space’, leveraging the emerging state identification architecture (architecture schematic: Fig. 7A; known stress states: D, NE, and D+NE; unknown stress states: H-D, H-NE, and H-D+NE). Known and unknown stress states generally occupied mutually exclusive regions of the ‘cardiovascular latent space’ across all folds at ~85% accuracy (folds 1-10 = panels **A** – **J**, respectively; region boundary: black dashed line, determined using a linear support vector machine; separation accuracy shown above each plot). These results demonstrate the ability to robustly increase interpretability and accurately visualize known and new unknown emerging stress states.

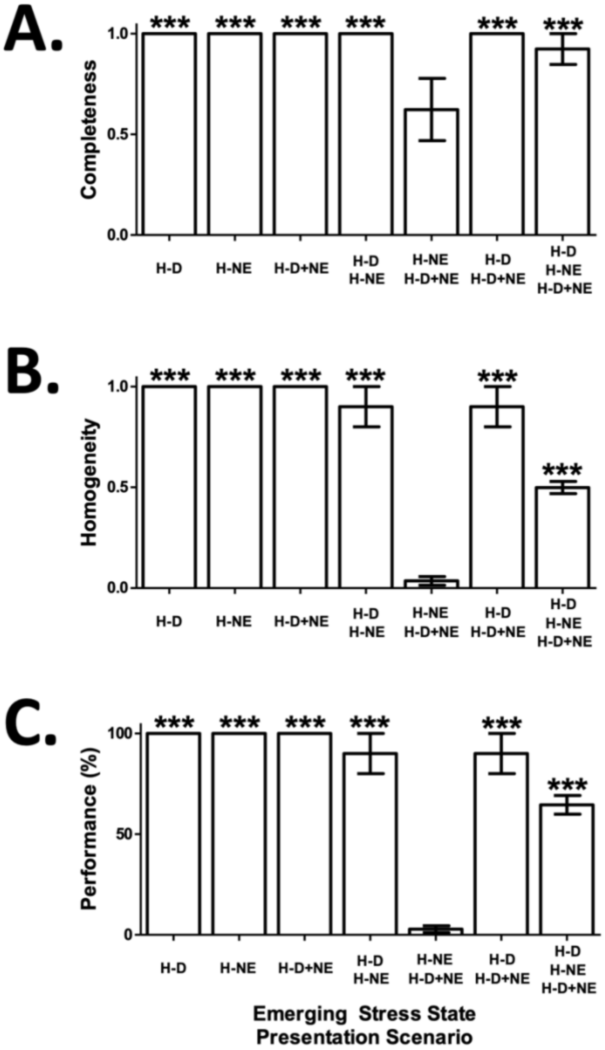

<u>Supplemental Figure S9. Emerging Cardiovascular Stress State Detection Performance Metrics Across All 7 Presentation Scenarios (related to Fig. 7C).</u> We performed emerging stress state detection across the 7 emerging stress state presentation scenarios (x-axes), and present well studied correlates of unsupervised clustering performance (*59*) including completeness (**A**), homogeneity (**B**), and performance (V-measure * 100%, **C**). Across metrics, almost all presentation scenarios performed well above chance performance levels (*** = different from chance at $p<0.001$). Data presented are mean ± SEM.

Supplemental Movie S1. **Increasing AI Interpretability: Visualizing Known and Unknown Cardiovascular Stress States in the ‘Cardiovascular Latent Space’.** We combined the LSTM-AE hidden layer with UMAP to create an interpretable visualization of known and unknown stress states (known stress states: D, NE, and D+NE; unknown stress states: H-D, H-NE, and H-D+NE; data shown is representative of a given fold; 36 total observations). The overall technique transformed the uninterpretable LSTM-AE hidden layer profile (256 dimensions, left panel of movie) into an interpretable representation (2-dimensional, right panel of movie) for a given stress state observation. Importantly, known (bottom set of points) and unknown (top set of points) stress states consistently generally occupied mutually exclusive regions of the 2 dimensional ‘cardiovascular latent space’. Furthermore, points within the known and unknown stress state regions formed separate clusters, indicating that the ‘cardiovascular latent space’ might also contain information relevant for identifying stress state subtypes, beyond just known and unknown states (see Fig. 7C for unsupervised dissociation of unknown stress state types). These results demonstrate a robust technique to visualize complex data and increase the interpretability of decoders and algorithm processes.
